# Predicting Response to Pro‐Cognitive Interventions in Mood Disorders: A Systematic Review by the International Society for Bipolar Disorders Targeting Cognition Task Force

**DOI:** 10.1111/acps.70038

**Published:** 2025-10-13

**Authors:** Dimosthenis Tsapekos, Michail Kalfas, Johanna M. Schandorff, Caterina del Mar Bonnin, Christopher R. Bowie, Vicent Balanzá‐Martínez, Katherine E. Burdick, Andre F. Carvalho, Annemieke Dols, Katie Douglas, Peter Gallagher, Gregor Hasler, Lars V. Kessing, Hanne L. Kjærstad, Beny Lafer, Kathryn E. Lewandowski, Carlos López‐Jaramillo, Anabel Martinez‐Aran, Roger S. McIntyre, Richard J. Porter, Scot E. Purdon, Ayal Schaffer, Paul R. A. Stokes, Tomiki Sumiyoshi, Ivan J. Torres, Tamsyn E. Van Rheenen, Lakshmi N. Yatham, Jeff Zarp, Allan H. Young, Eduard Vieta, Kamilla W. Miskowiak

**Affiliations:** ^1^ Centre for Affective Disorders, Department of Psychological Medicine, Institute of Psychiatry, Psychology and Neuroscience King's College London London UK; ^2^ Neurocognition and Emotion Across Disorders of the Brain (NEAD) Centre Psychiatric Centre Copenhagen, Mental Health Services, Capital Region of Denmark Frederiksberg Denmark; ^3^ Department of Psychology University of Copenhagen Copenhagen Denmark; ^4^ Institute of Neuroscience, Hospital Clinic University of Barcelona, IDIBAPS, CIBERSAM Barcelona Spain; ^5^ Department of Psychology Queen's University Kingston Canada; ^6^ Hospital Clínic Universitari, Department of Medicine University of Valencia, INCLIVA, CIBERSAM Valencia Spain; ^7^ Department of Psychiatry Harvard Medical School Boston Massachusetts USA; ^8^ Department of Psychiatry Brigham and Women's Hospital Boston Massachusetts USA; ^9^ IMPACT Strategic Research Centre (Innovation in Mental and Physical Health and Clinical Treatment) Deakin University Geelong Australia; ^10^ Department of Psychiatry, UMC Utrecht Brain Center University Medical Center Utrecht Utrecht the Netherlands; ^11^ Department of Psychological Medicine University of Otago Christchurch New Zealand; ^12^ Translational and Clinical Research Institute, Faculty of Medical Sciences Newcastle University Newcastle‐upon‐Tyne UK; ^13^ Psychiatry Research Unit University of Fribourg Fribourg Switzerland; ^14^ Copenhagen Affective Disorder Research Centre (CADIC), Psychiatric Centre Copenhagen Copenhagen University Hospital Copenhagen Denmark; ^15^ Department of Clinical Medicine University of Copenhagen Copenhagen Denmark; ^16^ Bipolar Disorder Research Program, Institute of Psychiatry, Hospital das Clinicas, Faculdade de Medicina Universidade de São Paulo São Paulo SP Brazil; ^17^ Schizophrenia and Bipolar Disorder Program McLean Hospital Belmont Massachusetts USA; ^18^ Department of Psychiatry Research Group in Psychiatry Universidad de Antioquia Medellín Colombia; ^19^ Department of Psychiatry University of Toronto Toronto Ontario Canada; ^20^ Department of Psychiatry University of Alberta Edmonton Canada; ^21^ Department of Preventive Intervention for Psychiatric Disorders National Institute of Mental Health, National Center of Neurology and Psychiatry Tokyo Japan; ^22^ Department of Psychiatry University of British Columbia Vancouver Canada; ^23^ Department of Psychiatry University of Melbourne Carlton Australia; ^24^ Centre for Mental Health and Brain Sciences, Faculty of Health, Arts and Design Swinburne University Hawthorn Australia; ^25^ Centre for Mental Health, Division of Psychiatry, Brian Sciences Imperial College London London UK

**Keywords:** bipolar disorder, cognitive impairment, cognitive interventions, ISBD, major depressive disorder, mood disorders, response predictors, systematic review, task force

## Abstract

**Introduction:**

Major depressive disorder (MDD) and bipolar disorder (BD) are often associated with persistent cognitive deficits that impair psychosocial functioning. While pro‐cognitive interventions show promise, trial findings are inconsistent, potentially due to baseline factors influencing treatment response. This systematic review summarizes evidence on pre‐treatment characteristics associated with cognitive improvement and offers methodological recommendations.

**Methods:**

A systematic search was conducted in PubMed/MEDLINE, EMBASE, PsycINFO, and Cochrane Library from inception to February 28, 2025. Eligible studies included primary or secondary analyses of randomized controlled trials (RCTs) investigating predictors of cognitive response to pro‐cognitive interventions in MDD and/or BD. Two researchers independently conducted study selection and risk of bias assessments. Findings were synthesized qualitatively.

**Results:**

Forty studies (*N* = 3864) were identified, covering pharmacological treatments (k = 20; *N* = 2299), psychological therapies (k = 16; *N* = 1165), brain stimulation (k = 2; *N* = 168), and physical activity (k = 2; *N* = 232). Poorer baseline cognitive performance was the most consistent predictor of greater cognitive improvement, though the direction of the effect was not entirely uniform across all studies. Baseline depression severity showed no significant association with cognitive outcomes. Age, education, sex, IQ, diagnosis, and medication status were similarly non‐predictive. Risk of bias was high in 77% of studies, mainly due to deviations from specified outcomes, poor randomization processes, and inconsistent handling of missing data. Considerable heterogeneity in interventions, outcome measures, and sample characteristics limited replicability and precluded meta‐analysis.

**Conclusion:**

Poorer baseline cognition emerged as the most reliable predictor of greater cognitive improvement across interventions. More rigorous, well‐powered studies are needed to replicate these findings and identify robust predictors to guide personalized pro‐cognitive treatment approaches in mood disorders.

**Summary**

- Significant outcomes
  ○ Poorer baseline cognitive performance was the strongest predictor of post‐treatment cognitive improvement, although the direction of the effect was not uniform.
  ○ Baseline depressive symptom severity was not significantly associated with post‐treatment cognitive change across studies.
  ○ In contrast, age, education, sex, IQ, diagnosis, and medication status showed no consistent association with treatment‐related cognitive outcomes.
- Limitations
  ○ Risk of bias evaluation indicated some or high risk of bias for the majority (77%) of the studies.
  ○ Key concerns were outcome deviations, poor randomization, and inconsistent handling of missing data.
  ○ Substantial heterogeneity across studies contributed to limited replicability and did not allow for a meta‐analytic approach.

## 1 Introduction

A significant proportion of individuals with major depressive disorder (MDD) and bipolar disorder (BD) display cognitive impairment across various domains, with moderate severity indicated by group means [1, 2, 3, 4] and more pronounced impairments in BD [5, 6, 7]. Recent clustering studies reveal considerable heterogeneity within both disorders, identifying at least three cognitive subgroups ranging from intact to severely impaired profiles [8, 9].

Cognitive deficits often persist beyond acute mood episodes [10, 11], impeding functional recovery and occupational functioning [12, 13, 14, 15, 16] and exacerbating the socio‐economic burden of mood disorders [17]. Deficits are also linked to worse illness trajectories, including increased risk of recurrence [18, 19], hospitalization [20], poorer treatment outcomes [21, 22], and lower quality of life [23, 24].

Recognizing cognition as a core feature has driven the search for pro‐cognitive treatments, leading to numerous clinical trials. The ISBD Targeting Cognition Task Force has reviewed randomized controlled trials (RCTs) assessing pharmacological, psychological, and neuromodulation interventions across 57 RCTs [25, 26, 27]. Cognitive remediation (CR) therapies showed the most promising, though preliminary, effects. Among pharmacological agents, modafinil and erythropoietin (EPO) benefited both MDD and BD [25, 26, 27]; mifepristone and lurasidone improved cognition in BD, while vortioxetine showed benefits in MDD. For neuromodulation, repetitive transcranial magnetic stimulation (rTMS) showed no cognitive effects, while transcranial direct current stimulation (tDCS) indicated some improvements in MDD [26]. Despite some promising findings, evidence remains preliminary, often limited by high or unclear risk of bias [25, 26, 27], as also noted by other reviews [28, 29].

Broad methodological challenges limit the generalizability of findings. These issues, along with recommendations for improving future trials, have been extensively reviewed by the ISBD Targeting Cognition Task Force [27, 30]. A key barrier to progress is the limited understanding of pre‐treatment characteristics influencing response. Although factors such as age, symptom severity, and baseline cognition have been examined, findings are scarce and inconsistent. Identifying predictors of treatment response (and non‐response) could reduce outcome variability, optimize intervention delivery, and guide adaptations for specific patient subgroups.

This systematic review by the ISBD Targeting Cognition Task Force summarizes, for the first time, evidence from cognition trials examining differential treatment response according to pre‐treatment factors in mood disorders. Our aims are to: (a) identify baseline characteristics predicting response, (b) review methodologies used to study predictors, and (c) provide methodological guidance for future investigations into differential treatment response.

## 2 Materials and Methods

This systematic review was conducted and reported in line with the Preferred Reporting Items for Systematic Reviews and Meta‐Analyses guidelines (PRISMA 2020 statement; [31]). A protocol was registered on PROSPERO (CRD420250648711) prior to the initiation of the review.

### 2.1 Eligibility Criteria

Only studies investigating the relationship between one or more pre‐treatment/baseline factors and the response to interventions targeting cognition, as examined by one or more primary or secondary/exploratory objectively measured cognitive outcomes (i.e., measured with performance‐based neuropsychological tests), were considered for inclusion. Eligible studies: (a) were peer‐reviewed articles in a scientific journal, published in English, (b) reported a primary or secondary/post hoc analysis of an RCT, and (c) had a sample primarily consisting of adult participants with a mood disorder (MDD/BD) diagnosis (i.e., ≥ 50%) according to the Diagnostic and Statistical Manual of Mental Disorders [32] or the International Classification of Diseases [33]. Where necessary, authors were contacted to request either additional data or data specific to the mood disorder subsample. We excluded: (a) non‐RCT study designs (e.g., case series), (b) conference/meeting abstracts, reviews, and meta‐analyses, and (c) studies without objective cognitive outcomes or not examining pre‐treatment/baseline predictors of response. We also did not consider trials examining baseline factors only as covariates/confounders in their efficacy analysis or assessing post‐randomization factors (e.g., therapy duration) and early treatment changes as predictors of response.

### 2.2 Search Strategy

A comprehensive systematic search was conducted on PubMed/MEDLINE, EMBASE, PsycINFO (using the Ovid interface), and Cochrane Library databases from inception to the 28th of February 2025. In line with the PICO framework (Population, Intervention, Comparison, Outcome), our search strategy included the following main components: mood disorder, treatment, RCT, cognition, predictor. Multiple keywords for each component were combined using ‘AND’ and ‘OR’ clauses. The search syntax is presented in detail in Supporting Information. To identify any additional eligible papers, the reference lists of included studies, previous reviews, and relevant publications in the field were hand‐searched.

### 2.3 Study Selection

The study selection process was performed independently by two authors (DT and MK) blinded to each other's selections, using the Rayyan open‐source review management software [34]. This first included screening titles and abstracts of all articles, then screening the full‐text papers of those retrieved as potentially eligible. Percent agreement between the two authors was 85% for the initial screening (corresponding to 0.7 Cohen's *kappa*) and > 90% for the full‐text screening (corresponding to > 0.82 Cohen's *kappa*). Disagreements were discussed until reaching consensus and, when needed, were resolved by the senior author (KWM).

### 2.4 Data Extraction

Data were extracted by DT and cross‐checked by MK. The following information was extracted from included studies: authors and year of publication, country, study design, sample size details, baseline mood state, basic demographic and clinical characteristics, treatment and control condition main features, cognitive outcomes and corresponding assessment time points, predictors assessed, type of analysis, and main findings for the effect of the predictor on the outcomes. For studies reporting multiple time points, we adopted the time point specified as primary or otherwise the latest one.

Data synthesis was organized according to the type of predictors examined. A quantitative meta‐analysis was not undertaken due to substantial heterogeneity in intervention modalities and treatment outcomes between studies.

### 2.5 Risk of Bias Assessment

Risk of bias (RoB) within and across the included studies was assessed independently by two authors (DT and JMS) using the Cochrane Collaboration's Tool for Assessing Risk of Bias 2.0 (RoB 2 tool; [35]). The RoB assessments focused on the analyses of cognitive outcomes in the included studies. Where necessary, we conducted additional searches in trial registries and published trial protocols to extract relevant details on study design and outcomes of interest. If these details were inadequately reported in primary or secondary publications, this was reflected in the RoB assessment. Scoring discrepancies were discussed until a consensus was reached and, if needed, were consulted with the senior author (KWM).

## 3 Results

**FIGURE 1:**
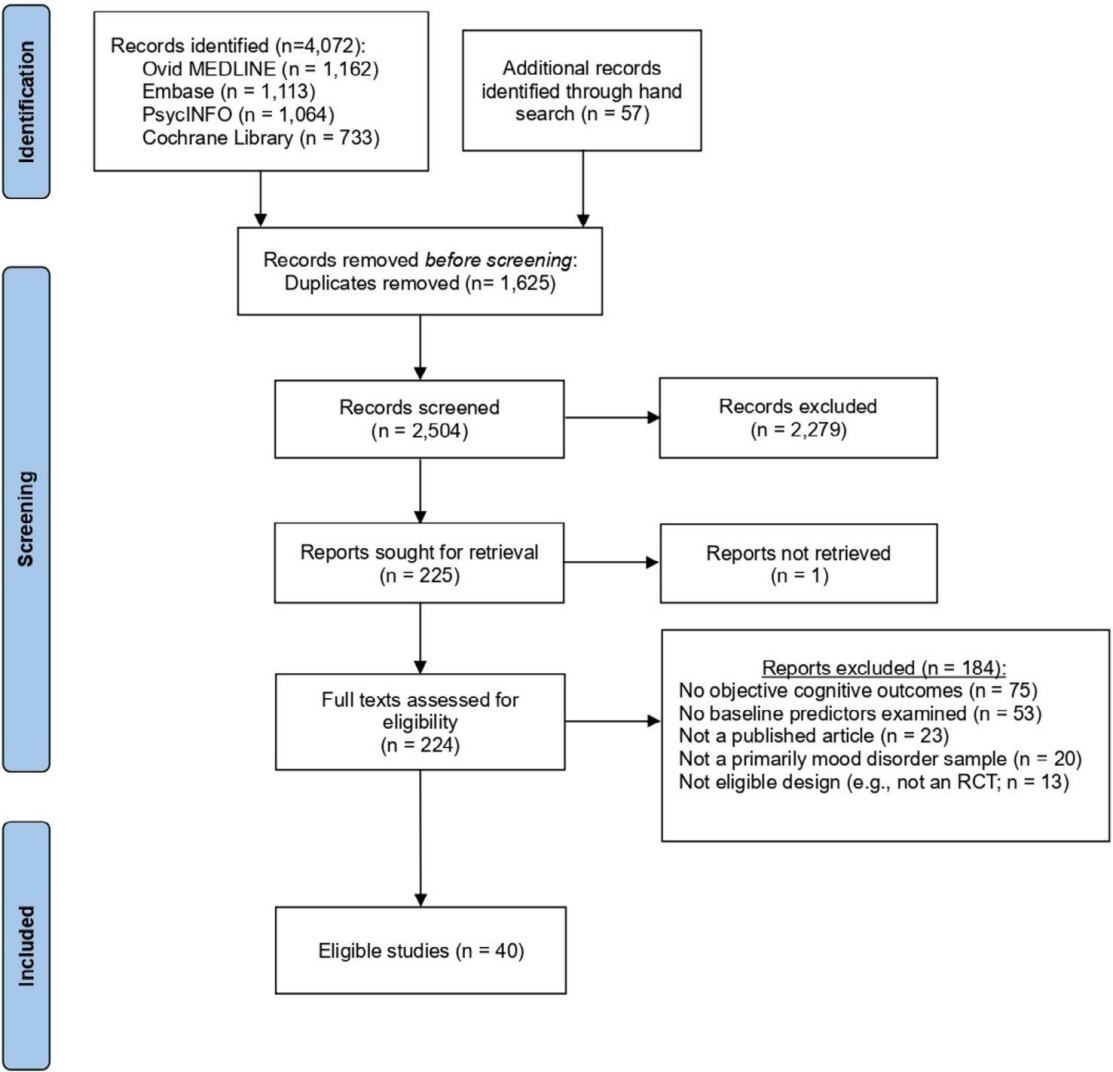
PRISMA flowchart.

Our systematic literature search, combined with additional hand searches, identified 2504 articles after removing duplicates. After initial screening, 224 were retrieved for full‐text evaluation. Of these, 184 reports were excluded with reasons provided, and 40 were included in the qualitative synthesis. A detailed PRISMA flowchart is presented in Figure 1. In total, studies reported on 3864 participants from 35 original RCTs assessing pharmacological or behavioral interventions targeting cognition in patients with mood disorders. A summary of the main study characteristics is displayed in Table 1.

**TABLE 1:** Characteristics of included studies.

Citation	Design	Setting	Participant characteristics								Mood disorder (%)	Intervention		Cognitive outcomes		
			Sample size			Age mean (SD)	Female %	Education years mean (SD)	Illness duration (years) mean (SD)	Mood state at baseline		Treatment	Control	Primary	Secondary/tertiary	Timepoint (weeks)
			*N*	Tx	C											
Pharmacological treatments																
Baune et al. 2021 [36]	RCT with cognition as exploratory outcome	Outpatients	119	59	60	45.3 (18.8)	58%	14.5 (2.8)	NR	Not remitted	MDD (100%)	Vortioxetine+ Celecoxib	Vortioxetine+ Placebo	NA	Codebreaker test (THINC‐it)	6
Burdick et al. 2012 [37]	RCT with cognition as the main outcome	Outpatients	45	21	24	44.5 (10.9)	63%	NR	NR	Remitted/partially remitted	BD (100%)	Pramipexole	Placebo	WM, PS, Att, EF, VeLM	NA	8
Dean 2012 [38]	Secondary analysis of RCT with cognition as the main outcome	Outpatients	46	21	25	45.5 (12.8)	60.1%	NR	10.1 (9.9)	Partially remitted	BD (100%)	*N*‐acetyl cysteine	Placebo	WM, VeLM, Att, EF	NA	24
Devanand et al. 2018 [39]	RCT with cognition as the main outcome	Outpatients	61	30	31	69.8 (8.3)	50.8%	15.4 (2.9)	NR	Partially remitted/not remitted	MDD (100%)	Donepezil	Placebo	ADAS‐Cog, VeLM	ViLM, VSF, Lang, Att, EF, PS	24
Kaser et al. 2017 [40]	RCT with cognition as the main outcome	Outpatients	60	30	30	45 (10.8)	61.7%	14 (2.8)	NR	Remitted	MDD (100%)	Modafinil	Placebo	Att, EF, SWM, ViLM	Att, EF, SWM, ViLM	1
Krause‐Sorio et al. 2021 [41]	Secondary analysis of RCT with cognition as exploratory outcome	Inpatients/outpatients	22	11	11	72.3 (6.9)	36.4%	16 (2.8)	NR	Partially remitted/not remitted	MDD (100%)	Escitalopram+ Memantine	Escitalopram+ Placebo	NA	DR, EF	24
Macoveanu et al. 2024 [42]	RCT with cognition as the main outcome	Outpatients	101	58	43	36.6 (12.3)	57.4%	14.6 (2.4)	13.1 (9)	Remitted	MDD & BD (100%)	Erythropoietin	Saline	Composite	Att	13
McIntyre et al. 2017 [43]	Secondary analysis of RCT with cognition as the main outcome	Inpatients/outpatients	598	402	196	45.9 (12.7)	66.2%	NR	NR	Not remitted	MDD (100%)	Vortioxetine 10 mg/20 mg	Placebo	PS, Att, VeLM, EF	NA	8
Miskowiak et al. 2020 [44]	Secondary analysis of RCT with cognition as the main outcome	Outpatients	76	37	39	41 (11)	68.4%	15 (3.5)	19 (11)	Partially remitted/not remitted	MDD & BD (100%)	Erythropoietin	Saline	VeLM	NA	9
Miskowiak Rush, et al. 2016 [45]	Secondary analysis of RCT with cognition as the main outcome	Outpatients	79	39	40	NR	66.7%	NR	NR	Partially remitted/not remitted	MDD & BD (100%)	Erythropoietin	Saline	VeLM	NA	9
Murphy Jr. et al. 2013 [46]	RCT with cognition as exploratory outcome	Outpatients	216	99	117	NR	NR	NR	NR	Not remitted	MDD (100%)	Paroxetine^a^	Mirtazapine	NA	Att, VeLM, ViLM, EF	8
Osuji 2010 [47]	RCT with cognition as exploratory outcome	Outpatients	60	29	31	41.7 (8.8)	48.3%	NR	NR	Not remitted	BD (73.3%) MDD (26.7%)	Pregnenolone	Placebo	NA	VeLM, EF	8
Ott et al. 2016 [48]	Secondary analysis of RCT with cognition as the main outcome	Outpatients	79	40	39	41.9 (11.6)	69.6%	15.3 (3.1)	19.4 (11.2)	Partially remitted/not remitted	MDD & BD (100%)	Erythropoietin	Saline	Composite	Att, PS, WM, VeLM, VSF, EF	14
Pelton et al. 2016 [49]	Secondary analysis of RCT with cognition as the main outcome	Outpatients	18	11	7	66.7 (9.5)	61.1%	12.5 (4.7)	NR	Not remitted	MDD (83%)	Donepezil	Placebo	Att, VeLM, EF	NA	12
Soczynska et al. 2014 [50]	RCT with cognition as the main outcome	Inpatients/outpatients	38	19	19	38 (11.4)	50%	14.3 (2.9)	15.7 (10.5)	Not remitted	MDD (100%)	Bupropion XL	Escitalopram/bupropion XL^a^	WM, VeLM, ViLM	NA	8
Van Dyk et al. 2020 [51]	Secondary analysis of RCT with cognition as exploratory outcome	Inpatients/outpatients	90	45	45	71.8 (6.9)	53.3%	15.8 (2.6)	NR	Partially remitted/not remitted	MDD (100%)	Escitalopram+ Memantine	Escitalopram+ Placebo	NA	VeLM, ViLM, EF	24
Van Meter 2021 [52]	RCT with cognition as the main outcome	Outpatients	60	31	29	39.4 (13.3)	58%	NR	NR	Partially remitted/remitted	BD (100%)	Pramipexole	Placebo	Composite	Impulsivity, PS, Att, WM, VeLM, ViLM, EF, SC	12
Watson et al. 2012 [53]	RCT with cognition as the main outcome	Outpatients	60	30	30	48 (9.4)	46.7%	NR	NR	Not remitted	BD (100%)	Mifepristone	Placebo	SWM	VeLM, VWM, EF, Att, ViEM	7
Xue et al. 2024 [54]	Secondary analysis of RCT with cognition as the main outcome	Outpatients	452	307	145	70 (3.2)	65.7%	NR	NR	Not remitted	MDD (100%)	Vortioxetine, Duloxetine	Placebo	Composite	DSST, RAVLT	8
Young et al. 2004 [55]	RCT with cognition as the main outcome	Outpatients	19^b^	19	19	49 (11)	NR	NR	NR	Not remitted	BD (100%)	Mifepristone	Placebo	SWM & VeLM	VWM, Att, ViLM, EF	6
Psychological therapies																
Bonnin et al. 2016 [56]	Secondary analysis of RCT with cognition as the main outcome	Outpatients	188	56	132	39.9 (9.2)	59.6%	12.6 (3.8)	14.2 (9)	Remitted	BD (100%)	Functional Remediation	Psychoeducation/treatment as usual	ND	IQ, PS, WM, ViLM, EF, Att	24
Demant 2015 [57]	RCT with cognition as the main outcome	Outpatients	40	18	22	34 (7.4)	62.5%	15.8 (3.2)	NR	Partially remitted	BD (100%)	Cognitive Remediation	Standard treatment	VeLM	Att, EF, WM, PS	12
Douglas et al. 2022 [58]	RCT with cognition as the main outcome	Outpatients	68	34	34	35.6 (12.1)	62%	NR	14.8 (13.4)	Partially remitted/remitted	BD (61.8%) MDD (38.2%)	Cognitive Remediation	Interpersonal and Social Rhythm Therapy	Composite	PS, WM, VeLM, ViLM, EF	52
Jespersen et al. 2023 [59]	RCT with cognition as the main outcome	Outpatients	40	20	20	31.4 (12)	55%	13.7 (2.2)	NR	Partially remitted	BD (40%) MDD (10%)	Virtual Reality Cognitive Training	Treatment as usual	Composite (VR tasks)	EF, WM, PS, VeLM, Att	1
Listunova et al. 2020 [60]	Secondary analysis of RCT with cognition as the main outcome	Outpatients	38	38	N/A	45.1 (13.4)	76.3%	NR	10.7 (10.2)	Partially remitted	MDD (100%)	Cognitive Remediation	NA	Att composite	NA	7
Miskowiak et al. 2021 [61]	Secondary analysis of RCT with cognition as the main outcome	Outpatients	53	28	25	36.9 (12.6)	73.5%	13.9 (3)	15.1 (11.3)	Partially remitted/remitted	BD (100%)	Action‐Based Cognitive Remediation	Conversation group	EF	NA	10
Miskowiak et al. 2021 [61]	Secondary analysis of RCT with cognition as the main outcome	Outpatients	45	25	20	36.9 (12)	77%	13.4 (3)	15.6 (10.8)	Partially remitted/remitted	BD (100%)	Action‐based cognitive remediation	Conversation group	EF	NA	10
Mogensen et al. 2022 [62]	Secondary analysis of RCT with cognition as the main outcome	Outpatients	45	25	20	36.6 (12)	77%	13.4 (3)	15.6 (10.8)	Partially remitted/remitted	BD (100%)	Action‐Based Cognitive Remediation	Conversation group	EF	NA	10
Primavera et al. 2024 [63]	Secondary analysis of RCT with cognition as the main outcome	Outpatients	21	11	4	61.4 (3.1)	71.4%	NR	NR	Partially remitted	BD (100%)	VR Cognitive Remediation	Treatment as usual	ND	ViLM, VeLM, WM, Att, Lang, PS, EF	12
Sanchez‐Moreno 2017 [64]	Secondary analysis of RCT with cognition as exploratory outcome	Outpatients	99	33	66	40 (8.3)	47.5%	13 (3.83)	14 (9.5)	Remitted	BD (100%)	Functional Remediation	Psychoeducation/Treatment as usual	NA	IQ, PS, WM, ViLM, EF, Att	24
Semkovska and Ahern 2017 [65]	RCT with cognition as the main outcome	Outpatients	22	11	11	46.4 (8)	82%	11.8 (1.8)	NR	Remitted	MDD (100%)	Neurocognitive Remediation	Computer games	ND	PS, Att, WM, VeLM, ViLM, EF	5
	RCT with cognition as the main outcome	Outpatient/inpatients	46	23	23	NR	NR	NR	NR	Remitted/not remitted	MDD (100%)	Neurocognitive Remediation	Computer games	ND	PS, Att, WM, VeLM, ViLM, EF	5
Sole 2015 [66]	Secondary analysis of RCT with cognition as exploratory outcome	Outpatients	53	17	36	41.1 (8.1)	58.5%	13 (3.1)	15.3 (9.6)	Remitted	BD (100%)	Functional Remediation	Psychoeducation/treatment as usual	NA	IQ, PS, WM, ViEM, EF, Att	24
Strawbridge et al. 2024 [67]	Secondary analysis of RCT with cognition as the main outcome	Outpatients	44	23	21	43.7 (12.8)	70%	15.2 (2.2)	12.1 (8.9)	Remitted	BD (100%)	Cognitive remediation	Treatment as usual	Composite	WM, Att, VeLM, EF	25
Thomas et al. 2017 [68]	Secondary analysis of RCT with cognition as the main outcome	Outpatients	77	77	N/A	44.4 (11.8)	44.2%	13.3 (2.7)	23.7 (12.9)	Partially remitted	MDD and BD (59.7%)	Compensatory Cognitive Training	NA	ND	PS, Att, WM, L/M, EF, PrM, & Global Deficit Score	12
Tsapekos et al. 2022 [69]	Secondary analysis of RCT with cognition as the main outcome	Outpatients	80	40	40	42.2 (12.9)	71.3%	15.8 (2.4)	11.8 (8.8)	Remitted	BD (100%)	Cognitive Remediation	Treatment as usual	Composite	WM, Att, VeLM, EF	13
Twamley et al. 2019 [70]	RCT with cognition as exploratory outcome	Outpatients	153	77	76	43.7 (11.7)	43.1%	13.4 (2.8)	24.4 (14.6)	Partially remitted	MDD & BD (62.1%)	Compensatory Cognitive Training	Enhanced Supported Employment	NA	PS, WM, L/M, EF, Att, PrM	12
Wanmaker et al. 2015 [71]	RCT with cognition as exploratory outcome	Outpatients	98	49	49	47 (12)	48.9	NR	NR	Remitted/partially remitted/not remitted	MDD (64.3%)	Working memory training	Bogus training	NA	WM	4
Brain stimulation techniques																
McClintock et al. 2020 [72]	RCT with cognition as the main outcome	Outpatients	120	61	59	48 (14.7)	52.5%	16.4 (6.7)	NR	Not remitted	MDD (70%) BD (30%)	High‐dose Transcranial Direct Current Stimulation (tDCS)	Low‐dose tDCS	ND	VeLM, Att, WM, PS, EF	4
Nadeau et al. 2014 [73]	Secondary analysis of RCT with cognition as the main outcome	Outpatients	48	34	14	46.6 (14.2)	60.4%	15.8 (2.4)	NR	Not remitted	MDD (100%)	Repetitive transcranial Magnetic Stimulation (rTMS)	Sham rTMS	ND	Lang, VSF, EF, VeLM, Att	12
Physical activity interventions																
Buschert et al. 2019 [74]	RCT with cognition as the main outcome	Inpatients	30	15	15	47.4 (7.7)	63.3%	13 (2.3)	NR	Not remitted	MDD (100%)	Physical activity	Active control (Occupational or art therapy)	ND	Att, WM, VeLM, EF	4
Hoffman et al. 2008 [75]	Secondary analysis of RCT with cognition as the main outcome	Outpatients	202	104	98	51.7 (7.6)	75.7%	15.8 (2.7)	NR	Not remitted	MDD (100%)	Aerobic Exercise	Sertraline/Placebo	ND	PS, WM, Att, VeLM, EF	16

*Note:* ^a^Both medications were examined as active treatments using a parallel design; ^b^Mifepristone was examined as an active treatment versus placebo using a cross‐over design.

Abbreviations: ADAS‐Cog: alzheimer's disease assessment scale‐cognitive subscale; Att: attention; BD: bipolar disorder; C: control; DR: delayed recall; DSST: digit symbol substitution test; MDD: major depressive disorder; EF: executive function; Lang: language; L/M: learning and memory; NA: not applicable; ND: not defined; NR: not reported; PrM: prospective memory; PS: processing speed; RAVLT: rey auditory verbal learning test; RCT: randomised controlled trial; SC: social cognition; SWM: spatial working memory; Tx: treatment; VeLM: verbal learning and memory; ViLM: visual learning and memory; VR: virtual reality; VSF: visual specific functioning; WM: working memory.

### 3.1 Study Characteristics

Most studies were conducted in Europe (k = 20), 12 in the USA, two in Australia, one in Canada, one in New Zealand, and four across multiple countries. Most of the included articles reported a primary (k = 14) or secondary analysis of an RCT (k = 17) with cognition as the main outcome, while fewer were either primary (k = 5) or secondary (k = 4) RCT analyses with cognition as an exploratory outcome. The mean sample size was 97 participants (SD = 110; range: 18–598), with a treatment group mean of 53 (SD = 73; range: 11–402) and a control group mean of 44 (SD = 41; range: 0–196). On average, pharmacological studies had a larger sample size (*N* = 115) than non‐pharmacological ones (*N* = 78).

### 3.2 Sample Characteristics

Participants had a mean age of 44.8 years (SD = 9.5; range: 31.4–72.3; k = 38), were mostly women (mean = 58.6%; SD = 10.4; range: 36.4%–82%; k = 38), with 14.4 years of education (SD = 3.1; range: 11.8–16.4; k = 26), and an average illness duration of 15.4 years (SD = 10.7; range: 10.1–24.4; k = 16). Across studies, the percentage of participants with a mood disorder diagnosis was 96% (SD = 13; range: 50–100%). Sixteen studies included participants with MDD, 14 included participants with BD, and 10 included mixed samples with both diagnoses. Mood state was balanced between studies recruiting remitted and/or partially remitted participants (k = 23) and those recruiting partially and/or not remitted participants (k = 17), with non‐pharmacological studies being over‐represented in the former and pharmacological studies more prevalent in the latter category.

### 3.3 Treatment Characteristics

Twenty studies investigated predictors of response to pharmacological treatments (*N* = 2299), 16 studies investigated predictors of response to psychological therapies (*N* = 1165), two studies investigated predictors of response to brain stimulation techniques (*N* = 168), and two investigated physical activity interventions (*N* = 232).

### 3.4 Control Characteristics

Of the 20 pharmacological studies, 18 used an inactive substance (e.g., placebo, saline) as the comparator, and two tested active compounds in both arms. For non‐pharmacological studies, 12 had an active control condition (e.g., computer games, psychoeducation), six had a non‐active control condition (e.g., standard treatment, treatment as usual), and two involved no control group participants in their secondary analysis.

### 3.5 Risk of Bias Assessment

**FIGURE 2:**
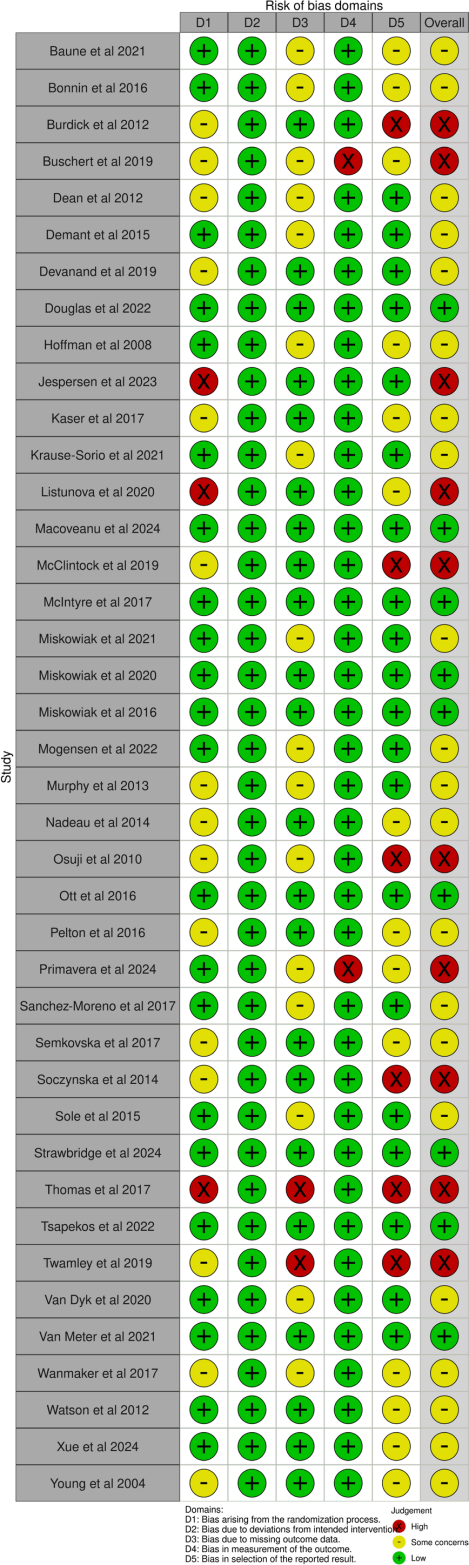
Risk of bias evaluations.

Figure 2 presents the RoB evaluations. Low RoB was detected in 22.5% of the studies, while some concerns and high RoB were found in 52.5% and 25% of the studies, respectively. The primary methodological concerns were lack of adherence to pre‐specified outcomes and analyses (15% high risk; 35% some concerns), limited details on or lack of randomization (7.5% high risk; 37.5% some concerns), and suboptimal handling of missing outcome data (5% high risk; 40% some concerns). In general, fewer pharmacological studies were of high RoB (15%) compared to non‐pharmacological studies (35%). Overall, studies often had methodological challenges regarding statistical analyses, which were most pronounced in non‐pharmacological studies.

### 3.6 Predictors of Treatment Response

Twenty‐three studies (58%) examined one variable as a predictor of response to a cognitive intervention, 10 studies (25%) assessed two predictors, and seven studies (17%) evaluated multiple predictors. Following a previously published approach [76], predictors of response were grouped into five categories: demographic, cognitive/functional, biological, clinical/illness‐related, and psychological. To facilitate results aggregation and interpretation, closely related predictors were grouped together under the same type. Key findings for each predictor type are summarized per category in Table 2.

**TABLE 2:** Predictors of cognitive outcomes identified across studies.

Predictors	# examined	Study	Analysis	Association with cognitive outcomes	Significant findings
Demographic					
Age	6	Hoffman et al. 2008 [75]	Linear regression	Νο	/
		Listunova et al. 2020 [75]	Subgroup comparison (means/frequencies)	No	/
		Nadeau et al. 2014 [73]	Linear regression	No	/
		Primavera et al. 2024 [63]	Non‐parametric regression	No	/
		Thomas et al. 2017 [68]	Linear regression	Yes	Younger age predicted greater improvement in category fluency for CCT (control not examined).
		Tsapekos et al. 2022 [69]	Linear regression (moderation)	No	/
Education	4	Soczynska et al. 2014 [50]	Linear regression	Yes	More education years predicted greater improvement in immediate verbal memory across groups.
		Buschert et al. 2019 [74]	Linear regression (moderation)	No	/
		Listunova et al. 2020 [60]	Subgroup comparison (means/frequencies)	No	/
		Tsapekos et al. 2022 [69]	Linear regression (moderation)	No	/
Sex	3	Listunova et al. 2020 [60]	Subgroup comparison (means/frequencies)	No	/
		Nadeau et al. 2014 [73]	Linear regression	No	/
		Tsapekos et al. 2022 [69]	Linear regression (moderation)	No	/
Premorbid IQ	2	Listunova et al. 2020 [60]	Subgroup comparison (means/frequencies)	No	/
		Tsapekos et al. 2022 [69]	Linear regression (moderation)	No	/
Relationship status	1	Listunova et al. 2020 [60]	Subgroup comparison (means/frequencies)	No	/
Housing situation	1	Listunova et al. 2020 [60]	Subgroup comparison (means/frequencies)	No	/
Cognitive/functional					
Baseline cognition	10	Devanand et al. 2018 [39]	Linear mixed models (subgroups)	No	/

Kaser et al. 2017 [40]		Non‐parametric correlation	Yes	Baseline cognitive performance correlated positively with post‐treatment performance (but not change scores) in multiple domains across groups.

Miskowiak, Rush, et al. 2016 [45]		Logistic regression	Yes	Memory dysfunction predicted achieving memory improvement in EPO but not placebo.

Ott et al. 2016 [48]		Logistic regression	Yes	Cognitive dysfunction predicted achieving cognitive improvement in EPO but not placebo.

Bonnin et al. 2016 [56]		Linear regression (selected subsample)	Yes	Cognitive impairment predicted improvement in verbal memory for FR group vs. TAU, but not psychoeducation.

Jespersen et al. 2023 [59]	Linear correlation	Yes	Lower cognitive performance predicted greater improvement in training group (control not examined).

Listunova et al. 2020 [60]	Subgroup comparison (means/frequencies)	No	/

Miskowiak et al. 2021 [61]	Linear regression (moderation)	No	/

Nadeau et al. 2014 [73]	Linear regression	Yes	Higher executive function performance predicted greater executive function improvement in across groups.

Tsapekos et al. 2022 [69]	Linear regression (moderation)	Yes	Lower cognitive performance predicted greater global cognition & verbal memory improvement across groups.
Baseline cognitive complaints	5	Ott et al. 2016 [48]	Logistic regression	No	/
		Listunova et al. 2020 [60]	Subgroup comparison (means/frequencies)	No	/
		Miskowiak Rush, et al. 2016 [45]	Logistic regression	Yes	More subjective cognitive difficulties predicted achieving memory improvement in EPO but not placebo.
		Miskowiak et al. 2021 [61]	Linear regression (moderation)	No	/
		Tsapekos et al. 2022 [69]	Linear regression (moderation)	No	/
Baseline functional capacity	2	Macoveanu et al. 2024 [42]	Linear mixed models (subgroups)	No	/
		Listunova et al. 2020 [60]	Subgroup comparison (means/frequencies)	No	/
Employment status	2	McIntyre et al. 2017 [43]	Subgroup comparison (linear regression)	Yes	Effects of vortioxetine vs. placebo on cognition were more pronounced for working subgroup vs. entire sample.
		Listunova et al. 2020 [60]	Subgroup comparison (means/frequencies)	No	/
Employment type	1	McIntyre et al. 2017 [43]	Subgroup comparison (linear regression)	Yes	Effects of vortioxetine vs. placebo on cognition were more pronounced for ‘professionals’ vs. working subgroup.
Biological					
Neuroimaging markers (structural)	9				
Cortical volume/thickness	5	Devanand et al. 2018 [39]	Linear mixed models (moderation)	No	/
		Miskowiak et al. 2020 [44]	Linear regression (moderation)	Yes	Larger left precentral gyrus surface area predicted greater improvement in verbal memory for EPO vs. placebo.
		Mogensen et al. 2022 [62]	Linear regression (moderation)	Yes	Less dPFC thickness predicted greater improvement in executive function for ABCR vs. control.
		Mogensen et al. 2022 [62]	Linear regression (moderation)	Yes	Smaller superior temporal volume predicted greater improvement in executive function for ABCR vs. control.
		Mogensen et al. 2022 [62]	Linear regression (moderation)	No	/
Hippocampal volume/asymmetry	3	Devanand et al. 2018 [39]	Linear mixed models (moderation)	No	/
		Miskowiak et al. 2020 [44]	Linear regression (moderation)	Yes	More rightward hippocampal asymmetry predicted greater improvement in verbal memory for EPO vs. placebo.
		Miskowiak et al. 2020 [44]	Linear regression (moderation)	No	/
White matter hyperintensities	1	Devanand et al. 2018 [39]	Linear mixed models (moderation)	Yes	High (vs low) white matter lesions volume predicted greater decline in general cognition and immediate verbal memory for donepezil vs. placebo.
Genetic factors	5				
ApoE genotype	1	Devanand et al. 2018 [39]	Linear mixed models (moderation)	No	/
BDNF genotype	2	McClintock et al. 2020 [72]	Linear regression (moderation)	Yes	BDNF Val66Met Val/Val genotype predicted greater improvement in verbal memory for high vs. low dose tDCS.
		Murphy Jr. et al. 2013 [46]	Linear regression	No	/
COMT genotype	1	McClintock et al. 2020 [72]	Linear regression (moderation)	Yes	COMT Val158Met Val/Val genotype predicted greater decline in verbal fluency for high vs. low dose tDCS.
CREB1 genotype	1	Murphy Jr. et al. 2013 [46]	Linear regression	No	/
Blood‐based markers	3				
Inflammatory cytokines and growth factors	2	Van Dyk et al. 2020 [51]	Linear regression	Yes	Higher pro‐inflammatory cytokine levels predicted greater decline in executive function for placebo vs. memantine.
		Strawbridge et al. 2024 [67]	Linear regression (moderation)	No	/
C‐reactive protein	1	Baune et al. 2021 [36]	Linear regression (moderation)	No	/
Neuroimaging markers (functional)	2				
[18F] FDDNP cortical binding	1	Krause‐Sorio et al. 2021 [41]	Non‐parametric regression	Yes	Higher frontal [18F] FDDNP binding predicted greater improvement in executive function across groups.
dPFC activation	1	Miskowiak et al. 2021 [61]	Linear regression (moderation)	Yes	More dPFC hypo‐activity during a WM task predicted greater executive function improvement in ABCR vs. control.
Hormonal markers	2				
Cortisol and progesterone	1	Watson et al. 2012 [53]	Non‐parametric correlation	No	/
Cortisol	1	Young et al. 2004 [55]	Non‐parametric correlation	Yes	Baseline cortisol levels correlated with improvement in spatial working memory for mifepristone vs. placebo.
Baseline olfactory capacity	2	Devanand et al. 2018 [39]	Linear mixed models (moderation)	No	/
		Pelton et al. 2016 [49]	Linear regression	Yes	Low olfactory capacity predicted greater improvement in verbal memory across groups.
Handedness	1	Nadeau et al. 2014 [73]	Linear regression	No	/
Stimulation laterality	1	Nadeau et al. 2014 [73]	Linear regression	No	/
Clinical/Illness‐related					
Baseline depression severity	9	Dean et al. 2012 [38]	Non‐parametric correlation	No	/
		Devanand et al. 2018 [39]	Linear mixed models (moderation)	No	/
		Buschert et al. 2019 [74]	Linear regression (moderation)	No	/
		Hoffman et al. 2008 [75]	Linear regression	Νο	/
		Listunova et al. 2020 [60]	Subgroup comparison (means/frequencies)	No	/
		Nadeau et al. 2014 [73]	Linear regression	No	/
		Sanchez‐Moreno et al. 2017 [64]	Linear regression (selected subsample)	No	/
		Tsapekos et al. 2022 [69]	Linear regression (moderation)	No	/
		Wanmaker et al. 2015 [71]	Linear regression (moderation)	No	/
Baseline medication use	5	Osuji et al. 2010 [47]	Subgroup comparison (linear regression)	No	/
		Van Meter et al. 2021 [52]	Linear mixed models	No	/
		Demant et al. 2015 [57]	Linear regression (subgroups)	No	/
		Listunova et al. 2020 [60]	Subgroup comparison (means/frequencies)	No	/
		Tsapekos et al. 2022 [69]	Linear regression (moderation)	No	/
Baseline remission status	4	Burdick et al. 2012 [37]	Subgroup comparison (linear regression)	Yes	Euthymic clinical status predicted greater cognitive improvement for pramipexole vs. placebo.
		Demant et al. 2015 [57]	Linear regression (subgroups)	No	/
		Listunova et al. 2020 [60]	Subgroup comparison (means/frequencies)	No	/
		Semkovska and Ahern 2017 [65]	Linear regression (moderation)	Yes	Depressed clinical status predicted greater improvement in visual/verbal memory for NCR vs. control and verbal working memory across groups.
Mood disorder diagnosis	3	Macoveanu et al. 2024 [42]	Linear mixed models (subgroups)	No	/
		Ott et al. 2016 [48]	Linear regression	No	/
		Douglas et al. 2022 [58]	Linear regression (moderation)	No	/
Age of onset	3	Xue et al. 2024 [54]	Linear regression (subgroups)	Yes	Depression onset later than 50 predicted greater improvement in global cognition across groups.
		Listunova et al. 2020 [60]	Subgroup comparison (means/frequencies)	No	/
		Tsapekos et al. 2022 [69]	Linear regression (moderation)	No	/
BD type diagnosis	3	Demant et al. 2015 [57]	Linear regression (subgroups)	No	/
		Sole et al. 2015 [66]	Linear regression (selected subsample)	No	/
		Tsapekos et al. 2022 [69]	Linear regression (moderation)	No	/
History of psychosis	2	Van Meter et al. 2021 [52]	Linear mixed models	No	/
		Tsapekos et al. 2022 [69]	Linear regression (moderation)	No	/
Illness duration	2	Listunova et al. 2020 [60]	Subgroup comparison (means/frequencies)	Yes	Improvers in attention after CR had shorter illness duration compared to non‐improvers.
		Tsapekos et al. 2022 [69]	Linear regression (moderation)	No	/
Number of mood episodes	2	Listunova et al. 2020 [60]	Subgroup comparison (means/frequencies)	No	/
		Tsapekos et al. 2022 [69]	Linear regression (moderation)	No	/
Number of hospitalisations	2	Listunova et al. 2020 [60]	Subgroup comparison (means/frequencies)	No	/
		Tsapekos et al. 2022 [69]	Linear regression (moderation)	No	/
Previous psychotherapies	2	Listunova et al. 2020 [60]	Subgroup comparison (means/frequencies)	No	/
		Tsapekos et al. 2022 [69]	Linear regression (moderation)	No	/
Childhood trauma	2	Listunova et al. 2020 [60]	Subgroup comparison (means/frequencies)	No	/
		Tsapekos et al. 2022 [69]	Linear regression (moderation)	No	/
Baseline anxiety severity	2	Tsapekos et al. 2022 [69]	Linear regression (moderation)	No	/
		Wanmaker et al. 2015 [71]	Linear regression (moderation)	No	/
Baseline (hypo)mania severity	1	Tsapekos et al. 2022 [69]	Linear regression (moderation)	No	/
Psychiatric diagnosis	1	Twamley et al. 2019 [70]	Linear regression (moderation)	Yes	Having a mood disorder diagnosis predicted greater improvement in attention across groups.
Current psychotherapy	1	Listunova et al. 2020 [60]	Subgroup comparison (means/frequencies)	No	/
Psychological					
Motivation	1	Listunova et al. 2020 [60]	Subgroup comparison (means/frequencies)	No	/
Anticipatory/consummatory pleasure	1	Listunova et al. 2020 [60]	Subgroup comparison (means/frequencies)	No	/
Therapy hope/expectations/scepticism	1	Semkovska and Ahern 2017 [65]	Linear correlation	No	/
Rumination	1	Wanmaker et al. 2015 [71]	Linear regression (moderation)	No	/

Abbreviations: [18F] FDDNP: 2‐(1‐{6‐[(2‐[fluorine18]fluoroethyl)(methyl)amino]‐2‐naphthyl}‐ethylidene)malononitrile; ABCR: action‐based cognitive remediation; APoE: apolipoprotein E; BD: bipolar sisorder; BDNF: brain‐derived neurotrophic factor; CCT: compensatory cognitive training; COMT: catechol‐o‐methyltransferase; CR: cognitive remediation; CREB1: cyclic AMP responsive element binding protein 1; dPFC: dorsal prefrontal cortex; EPO: erythropoietin; NCR: neurocognitive remediation; tDCS: transcranial direct current stimulation; TAU: treatment‐as‐usual; WM: working memory.

#### 3.6.1 Demographic Factors

Six demographic predictors were examined across eight studies (*N* = 534): age, education, sex, premorbid IQ, relationship status, and housing situation. Of the 17 instances when a demographic response predictor was tested, an association with cognitive improvement was only reported twice (12%), once for age and once for education, without any replication in different samples (Table 2).

Specifically, five of six studies found no association between age and cognitive improvement [60, 63, 69, 73, 75], while one reported that *younger* MDD and BD partially remitted outpatients benefited more than older ones in category fluency following compensatory cognitive training [68].

Three of the four studies found no influence of education [50, 60, 69, 74], while one study showed that higher education predicted greater verbal memory improvement after both bupropion XL and escitalopram treatment in depressed individuals with MDD [50].

No significant associations were found for sex [60, 69, 73], premorbid IQ [60, 69], relationship status, and housing situation [60].

#### 3.6.2 Cognitive and Functional Factors

Collectively, five types of cognitive/functional predictors were considered, including baseline objectively measured cognition, subjectively reported cognitive complaints, psychosocial functioning, employment status, and employment type. In 20 analyses across 12 studies (*N* = 1425), a significant association was found ten times (50%).

Objectively measured cognition at baseline was the most consistent predictor of post‐treatment cognitive changes (six of 10 studies), although the direction of this association was not uniform: five studies (83%) found that poorer baseline cognition predicted better outcomes [45, 48, 56, 59, 69], while one study found that better executive function predicted greater improvement in this domain [73] (Table 2). Specifically, two secondary RCT analyses reported that cognitively impaired MDD (depressed) and BD (partially remitted) outpatients were more likely to achieve a clinically relevant improvement in memory [45] and global cognition following treatment with EPO [48]. Importantly, the effect of baseline cognition was specific to participants receiving active treatment, indicating it was not merely due to regression to the mean. A similar association—where lower baseline cognition predicted greater treatment‐related improvement—was observed in two RCTs of cognitive remediation (CR) paradigms in remitted BD [69] and partially remitted patients with MDD or BD [59]. In a secondary analysis of an RCT of action‐based CR, a non‐significant trend (*p* = 0.08) also suggested that poorer baseline performance predicted greater executive function improvement in the active versus control groups [61]. A comparable effect was reported in a secondary analysis of a functional remediation (FR) trial [56]: while the original RCT found no significant cognitive effects, this analysis—focused on cognitively impaired participants—revealed greater post‐treatment verbal memory improvement following FR compared to treatment as usual.

In contrast to five studies linking poorer baseline cognition to greater intervention benefits, one rTMS study in depressed MDD outpatients found that better baseline executive function predicted greater improvement across groups [73]. This small study (*N* = 48) had the potential for selective outcome reporting (see Figure 2). Finally, one study in remitted MDD outpatients assessed the correlation between baseline and post‐intervention performance across the full sample, including both modafinil and placebo groups [40]. While a non‐specific positive association was observed—indicating that individuals with lower baseline scores also tended to score lower post‐intervention—the authors did not examine the relationship between baseline performance and cognitive *change* scores, nor whether this relationship was moderated by modafinil. As such, it remains unclear whether baseline performance was associated with cognitive change in response to the intervention.

Of the five studies investigating subjective cognitive complaints, only one found that greater self‐reported difficulties were weakly related to memory improvement in the active versus control groups [45], while four reported no significant effects [60, 61, 69].

Regarding psychosocial functioning, two studies found that this was not predictive of post‐treatment cognitive changes [42, 60]. Employment status and type were examined as response predictors in a large RCT comparing vortioxetine to placebo in depressed MDD participants [43]. Further, being employed and having a ‘professional’ based employment type predicted greater improvement in multiple cognitive outcomes compared to the entire sample and working subgroup, respectively. However, the effect of employment status was not replicated in a smaller CR study [60], rendering the evidence inconsistent.

#### 3.6.3 Neuroimaging and Biological Factors

Eight types of neuroimaging and biological markers were considered by 14 studies (*N* = 991). A significant association with cognitive changes following treatment was detected in 12 of 25 analyses (48%), primarily for structural neuroimaging markers (Table 2).

Cortical volume or thickness was identified as predictors of response by two studies: larger left precentral gyrus surface area moderated the effect of EPO versus placebo on verbal memory in a mixed sample of symptomatic and remitted mood disorder outpatients [44], while lower dPFC thickness and smaller superior temporal volume were associated with greater treatment‐specific executive function improvement in fully or partially remitted BD participants receiving action‐based CR [62]. Total hippocampal volume did not affect outcomes in two studies [39, 44], whereas more right‐ward hippocampal volume asymmetry (a marker of brain reserve) predicted greater verbal memory benefits for EPO‐treated patients [44]. Finally, more white matter hyperintensities were associated with greater cognitive decline for donepezil‐ versus placebo‐treated older MDD individuals [39].

Regarding functional neuroimaging markers, dorsal prefrontal cortex (dPFC) *hypoactivity* during a working memory task at baseline was associated with greater efficacy of CR on executive function [61]. Further, higher baseline [18F] FDDNP binding (a measure of brain amyloid and neurofibrillary tangle load) in the frontal lobe predicted more executive function benefits across participants treated with memantine or placebo adjunctive to escitalopram [41].

A study comparing high‐ and low‐dose tDCS in depressed outpatients found that Brain‐Derived Neurotrophic Factor (BDNF) Val66Met and Catechol‐O‐methyltransferase (COMT) Val158Met polymorphisms interacted with tDCS dose, resulting in greater improvements in verbal memory and fluency in the high‐dose group [72]. No other examinations of genetic factors showed significant findings [39, 46].

Other baseline biomarkers associated with post‐intervention cognitive changes included inflammatory cytokine levels [51], cortisol awakening response [55], and olfactory capacity [49]. However, for all three, there were also examinations showing no significant associations [36, 39, 53, 67], rendering these findings inconsistent.

#### 3.6.4 Clinical and Illness‐Related Factors

In this broad category, the relationship between 16 candidate predictors and treatment response on cognition was examined in 44 instances by 20 studies (*N* = 1881), but a significant association was only reported five times (11%). Notably, baseline depression severity did *not* moderate any post‐treatment cognitive improvement despite being the most intensively investigated clinical variable (Table 2). Likewise, treatment response was unrelated to baseline medication use, mood disorder diagnosis type, BD subtype, psychosis history, number of mood episodes or hospitalizations, baseline anxiety or (hypo)mania, childhood trauma, or previous/current psychotherapies (investigated in a few studies; Table 2).

Two of four studies examining the role of baseline remission status showed significant associations with cognitive improvements but in opposite directions (Table 2). In one trial testing pramipexole versus placebo in fully or partially remitted BD outpatients, the fully remitted subgroup experienced greater post‐treatment improvements compared to the full‐sample analysis [37]. In contrast, another trial found that depressive symptoms (vs. being in remission) at baseline predicted greater memory improvements for MDD participants receiving online CR [65].

A large secondary analysis found that elderly depressed outpatients with illness onset *after* age 50 showed greater post‐treatment gains in global cognition across vortioxetine, duloxetine, and placebo groups [54]. Finally, individual trials found better responses to psychological therapies in those with shorter illness duration [60] and mood disorders versus schizophrenia‐spectrum diagnoses [70], though these effects were not specific to the treatment groups.

#### 3.6.5 Psychological Factors

Four studies (*N* = 204) examined psychological predictors of cognitive outcomes in partially or fully remitted MDD outpatients, but none found significant associations. Motivation did not differ between attention improvers and non‐improvers after CR [60]. Further, therapy‐related hopes, expectations, and skepticism were unrelated to cognitive improvements [65], and baseline rumination did not moderate working memory training effects [71].

## 4 Discussion

This systematic review aimed to identify baseline factors associated with treatment response in primary or secondary analyses of RCTs on pro‐cognitive interventions for mood disorders. We identified 20 baseline factors predicting cognitive outcomes of pro‐cognitive interventions, but with limited replication and evidence convergence between studies. Of the factors considered, poorer cognitive function at baseline was the most consistent predictor of greater post‐treatment improvements. In contrast, depression severity and medication use consistently showed no significant associations with changes in post‐treatment cognitive outcomes. Other clinical and demographic variables, albeit less intensively investigated, also showed no consistent association with treatment response. Structural and functional differences in the prefrontal cortex, temporal lobe, and hippocampus were the most identified neuroimaging biomarkers of cognitive improvement, though examined in only a few studies. More than 75% of the included studies presented with some methodological concerns or a high risk of bias.

### 4.1 Implications and Recommendations

The association between poorer baseline cognition and greater treatment‐related cognitive improvement was the most consistently replicated finding in this review, reported in five of ten studies [45, 48, 56, 59, 69]. In contrast, four studies found no significant association, and one small rTMS study reported a non‐specific opposite effect, where better baseline executive function predicted improvement across both active and sham groups [73]. This may reflect practice effects, as individuals with executive impairments typically show reduced gains from repeated testing, whereas those with better cognition may improve simply due to retesting [77]. These observations highlight the need to examine baseline cognition specifically in relation to treatment effects, rather than interpreting overall cognitive change, which may be confounded by practice effects or regression to the mean. Despite this, only three of six studies tested for an interaction between baseline cognition and treatment arm (Table 3), all of which found a significant influence [25, 48, 56]. Analytical approaches and definitions of cognitive impairment varied (Table S1), reflecting heterogeneity in how baseline cognition was operationalised and assessed. Importantly, associations were found in studies both with and without pre‐screening for cognitive impairment.

**TABLE 3:** Methodological recommendations relevant to predictors of response to pro‐cognitive interventions by the International Society for Bipolar Disorders Targeting Cognition Taskforce.

Quick guide:
How can we enrich trials with cognitively impaired participants? Pre‐screen participants for *objective* cognitive impairments using a brief cognition screening tool (e.g., SCIP). Pre‐screen for either (i) *broad* cognitive impairments in trials for which a global cognitive composite is the primary outcome or (ii) *specific* deficits in a particular cognitive domain in studies of interventions with a purported specific cognitive target. Use a *different* cognitive test battery to (or a parallel version of) the cognitive test battery implemented as the primary outcome. To screen for impairment in a particular domain, use of several tests (rather than a single test) that tap into this domain.
2 What is a feasible threshold for cognitive impairment? ≥ 0.5 SD below the normative mean for a cognitive composite based on a screening tool. ≥ 1 SD below the normative mean on ≥ 2 single cognitive tests for a specific domain.
3 How can we establish impairment with reference to premorbid capacity? Administer a short, validated test of premorbid IQ (e.g., NART/TOPF). Adjust current cognitive performance for premorbid IQ by *subtracting* the standardized IQ score from standardized score of current global performance (e.g., SCIP z score minus NART/TOPF z score). As before, apply recommended thresholds for impairment to the adjusted score.
4 How can we examine the impact of baseline cognition in trials where cognition is not an inclusion criterion? Conduct a post hoc analysis to assess baseline cognition, either with or without adjusting for premorbid IQ, as a baseline factor influencing treatment efficacy on the primary cognitive outcome. Consider moderation analysis to ensure that significant effects are treatment‐related and specific to the active intervention.
5 How can we consolidate evidence on predictors of response to pro‐cognitive interventions? Disseminate both *positive* and *negative* associations between baseline variables and cognitive improvements. Combine datasets from trials testing the same or comparable interventions for adequately powered hypothesis‐driven analyses of predictors with initial evidence. If possible, adopt a broad composite score encompassing core domains (attention, processing speed, verbal memory, executive functions) as the primary cognitive outcome, using tests that are broadly equivalent to those included in the ISBD‐BANC [78].
6 Which mood and medication use criteria should be used to select trial participants? To rigorously minimise pseudospecificity in trials with cognition as the primary outcome, include partially or fully remitted participants. If full separation of cognitive and mood effects is not essential, consider including participants across the baseline mood spectrum to support recruitment, as depressive symptoms do not influence treatment response (see below for guidance on managing pseudospecificity). Monitor mood fluctuation during the trial to tackle engagement challenges. Allow concomitant medications; these do not seem to affect treatment response on cognition. Concomitant medications should be within the recommended doses, be carefully recorded and, if possible, be kept stable ahead of and during trial participation. Taper benzodiazepines to a maximum dose equivalent to 22.5 mg oxazepam/7.5 mg diazepam per day and restrict use of benzodiazepine and other hypnotics 6 h prior to cognitive testing.
7 How should ‘pseudospecificity’ be addressed? Include assessments of depressive and (hypo)manic symptoms during trial participation as covariates in the efficacy analysis of cognitive outcomes.
8 Neuroimaging assessments in treatment trials: why and how? If possible, implement neuroimaging assessments (e.g., pre‐ and post‐treatment) to investigate whether candidate pro‐cognitive interventions target functional and/or structural abnormalities that underlie cognitive impairments. Select areas/volumes/regions of interest based on strong hypotheses and/or existing evidence (even if pre‐clinical) to identify neuroimaging biomarkers associated with specific pro‐cognitive interventions.

This association between poorer baseline cognition and better treatment response extends findings from a prior review [22]. Further support comes from non‐randomised studies showing that individuals with greater cognitive deficits are more likely to benefit from pro‐cognitive interventions [79]. These results align with the hypothesis that lower baseline cognitive levels provide greater ‘room for improvement,’ while intact or mildly impaired individuals experience ceiling effects [30].

Taken together, these findings underscore the importance of pre‐screening for objective cognitive impairment, aligning with ISBD Taskforce recommendations [27, 30] (Table 3). Brief screening tools, such as the Screen for Cognitive Impairment in Psychiatry (SCIP; [80]), offering both global and domain scores, are recommended. Screening for domain‐specific impairment should involve more than one test per domain, and trials should avoid defining cognitive impairment by a single test as the primary outcome [27]. Based on prior recommendations, feasible thresholds include 0.5 SD below normative means for composite scores and 1 SD below the mean on two or more tests for domain scores [30]. Given that many individuals with mood disorders show no or only mild cognitive impairment [81], strict pre‐screening based on norms could impede recruitment [82]. An alternative is estimating illness‐related cognitive decline by comparing current performance to premorbid IQ, using discrepancy scores [83]. Tools like the National Adult Reading Test (NART; [84]) or Test of Premorbid Functioning (TOPF; [85]) can assist. Applying a predefined discrepancy threshold enables identifying individuals with relative impairments—those likely to benefit—while avoiding recruitment barriers [86]. In trials where cognition is a secondary outcome, post hoc analyses adjusting for premorbid IQ are recommended.

Unlike objective measures, baseline subjectively reported cognitive difficulties showed little association with objective cognitive outcomes, consistent with poor correspondence between subjective and objective cognition in mood disorders [87, 88]. This highlights that relying solely on self‐reports for trial inclusion is insufficient. Similarly, baseline medication use showed no consistent association with cognitive change when participants had stable medication regimens, supporting the continued inclusion of stably medicated participants in cognition trials [27].

Notably, baseline depressive symptom severity showed no impact on treatment‐related cognitive gains, despite varying study designs, sample sizes, interventions, and outcomes. Cognitive improvements thus appear largely independent of baseline mood, suggesting strict pre‐screening for remission may not be essential when cognition is the primary outcome. Although depressive symptoms differed across studies, mean baseline scores indicated mild symptoms overall (HDRS: M = 14, k = 6; BDI‐II: M = 21, k = 2; MADRS: M = 14, k = 1). This finding suggests an updating of earlier recommendations to restrict inclusion to fully or partially remitted patients [27], allowing inclusion of patients with slightly higher depressive symptom levels. However, when the aim is to target trait‐like cognitive deficits persisting during remission—and to separate cognitive from mood improvements—enrolling partially or fully remitted participants remains advisable. In trials where this distinction is less critical, mood fluctuations should be controlled for by including mood changes as covariates in analyses. Severe acute mood symptoms may impair engagement, especially with psychological interventions, reducing treatment effects [58]. Thus, a minimum degree of mood stability should be ensured prior to enrollment to support participation and interpretation of results.

Finally, identifying biomarkers of treatment response can clarify neurobiological mechanisms underlying cognitive improvements and highlight future intervention targets [30]. Several promising biomarkers were found, particularly structural and functional neuroimaging measures involving the prefrontal cortex, temporal cortex, and hippocampus. Although findings varied, this may reflect the distributed nature of cognitive processes. Most studies were grounded in strong theoretical frameworks about neural intervention effects. However, small samples testing multiple biomarkers likely increased type I error risk [89]. To improve insights into neural target engagement, neuroimaging should be hypothesis‐driven and focused on brain abnormalities linked to targeted cognitive impairments [27]. Nevertheless, the clinical utility of neuroimaging biomarkers remains limited due to accessibility and cost barriers.

### 4.2 Methodological Limitations and Considerations

A major limitation across the included studies was poor methodological quality, with 77% rated as having a high risk of bias. This was often due to cognition being examined as a secondary or exploratory outcome. Other key issues included a lack of prespecified primary outcomes, deviations from specified outcomes, limited reporting of randomisation procedures, and inadequate or missing handling of missing data—particularly in non‐pharmacological trials. Furthermore, studies frequently assessed multiple predictors and cognitive outcomes without correcting for multiple comparisons, increasing the risk of false positives. Conversely, small to moderate sample sizes may have led to false negatives due to insufficient statistical power. Although methodological limitations may thus introduce false positive or negative associations, this risk is reduced when predictors show consistent results across multiple studies. For example, baseline cognition demonstrated associations in most of 10 studies, whereas baseline depression severity showed none across nine, suggesting reliable patterns rather than random error.

Heterogeneity across studies—in intervention modalities, outcome measures, predictor assessments, analytical approaches, and sample characteristics (e.g., mood state at entry)—further limited replication and hindered robust conclusions. This diversity also precluded meta‐analysis. Nevertheless, qualitative synthesis offered preliminary insights into factors predicting response to pro‐cognitive interventions and informed recommendations for future research. Although publication bias was not formally assessed, the frequent reporting of non‐significant associations suggests a relatively low risk.

An important methodological consideration for future cognition trials is the approach to analyzing baseline predictors of response. Across the studies reviewed, we identified a range of methods, including correlation analyses, linear and logistic regression, subsample analyses, and subgroup comparisons (Table 2). While all are relevant, it is essential to determine whether observed associations are specific to the active treatment or reflect natural regression to the mean [90]. Moderation analysis—including an interaction term between treatment and baseline predictor—is recommended [91, 92], as it allows identification of treatment‐specific effects.

Another limitation was the potential confounding effects of concomitant pharmacotherapy, as most studies employed naturalistic medication management [93].

Finally, the limited and inconsistent efficacy of cognitive interventions themselves remains a critical challenge. Without a consistently strong treatment signal, the identification of robust predictors is inherently constrained. Addressing this will be essential for advancing precision approaches in cognition trials.

### 4.3 Future Directions

Future cognition trials should systematically examine and report baseline predictors of treatment response to expand the evidence base. While positive results are preferentially published [94], reporting of null findings is equally crucial to avoid bias and enhance understanding. The limited replication observed in this review likely reflects small sample sizes and exploratory study designs. Advancing the field requires large, adequately powered, hypothesis‐driven studies [76], with data sharing or pooling efforts needed to reliably estimate the effects of candidate predictors, such as baseline cognition.

To minimise variability, future studies should adopt standardised cognitive outcomes [95]. In line with previous recommendations, the primary outcome should ideally be a composite score covering core cognitive domains, unless a specific domain is targeted—where the use of multiple tests for that domain is advised [27]. Calculation of a g‐factor via Principal Component Analysis across the cognitive battery is also recommended to enhance sensitivity and comparability.

By strengthening methodological rigor and standardization, future trials can more reliably identify predictors of cognitive improvement and move the field closer to precision‐guided interventions for individuals with mood disorders.

### 4.4 Conclusions

This systematic review provides initial evidence on baseline factors influencing treatment response and outcome variability in cognition trials for mood disorders. We identified five broad predictor categories, most of which showed non‐significant findings, conflicting associations, or lacked replication. Substantial sources of bias were also evident across studies. Baseline objective cognition emerged as the most consistent predictor of response to pro‐cognitive interventions, although not uniformly across all studies. While preliminary, these findings support several methodological recommendations previously issued by the ISBD Targeting Cognition Taskforce (summarized in Table 3) and offer guidance for harmonizing future research. Improving the methodological quality of cognition trials remains a key priority. To reliably estimate the effects of baseline cognition and other candidate predictors, adequately powered, hypothesis‐driven analyses in large cohorts or merged datasets are essential. A clearer understanding of pre‐treatment factors may ultimately enable more efficient and effective application of pro‐cognitive strategies for individuals with mood disorders.

## Ethics Statement

The authors have nothing to report.

## Conflicts of Interest

Christopher R. Bowie has been a consultant for Boehringer Ingelheim. He receives book royalties from Oxford University Press. He has received in‐kind research user accounts from Scientific Brain Training Pro. Kate Burdick received honorarium and grant funding for her leadership role in the Breakthrough Discoveries for thriving with Bipolar Disorder (BD^2^) and she serves on scientific advisory boards for Suven Life Sciences and Alto Neuroscience. Katie Douglas reports using software provided free of charge by Scientific Brain Training Pro for Cognitive Remediation trials. Gregor Hasler reports speaker/consultant fees from Janssen, Lundbeck, OM Pharma, Otsuka, Sanofi, Schwabe, Servier, Sunovion and Takeda. Beny Lafer has received grants from Coordenação de Aperfeiçoamento de Pessoal de Nível Superior (CAPES), Conselho Nacional de Desenvolvimento Científico e Tecnólógico (CNPq) and the Baszucki Brain Research Fund. Michail Kalfas has received honoraria from Neurocentrx Pharma Ltd. and is supported by the National Institute for Health Research (NIHR) Biomedical Research Centre. Hanne L. Kjærstad has received honoraria from Lundbeck. Anabel Martinez‐Aran receives support by d'Economia i Coneixement (2021 SGR 01128), the Centro de Investigación Biomédica en Red de Salud Mental‐CIBERSAM and the Centres de Recerca de Catalunya‐CERCA Programmme. Roger S. McIntyre has received research grant support from CIHR/GACD/National Natural Science Foundation of China (NSFC) and the Milken Institute; speaker/consultation fees from Lundbeck, Janssen, Alkermes, Neumora Therapeutics, Boehringer Ingelheim, Sage, Biogen, Mitsubishi Tanabe, Purdue, Pfizer, Otsuka, Takeda, Neurocrine, Neurawell, Sunovion, Bausch Health, Axsome, Novo Nordisk, Kris, Sanofi, Eisai, Intra‐Cellular, NewBridge Pharmaceuticals, Viatris, Abbvie, Atai Life Sciences. Dr. Roger McIntyre is a CEO of Braxia Scientific Corp. Kamilla W. Miskowiak is supported by a European Research Council (ERC) Consolidator Grant. She has received honoraria from Allergan, Gedeon Richter, Angelini and Lundbeck in the past 3 years. Richard J. Porter reports use of computer software for research –provided at no cost by SBT‐pro and funding for travel to educational meetings by Lundbeck and Servier. Ivan J. Torres has received consulting fees from Boehringer Ingelheim (Canada). Tamsyn E. Van Rheenen is supported by an Al and Val Rosenstrauss Fellowship from the Rebecca L Cooper Medical research Foundation and a Dame Kate Campbell Fellowship from the University of Melbourne. Paul R.A. Stokes reports grant funding from the Medical Research Council UK, National Institute for Health and Care Research (NIHR), H. Lundbeck A/S and King's Health Partners, funding from NIHR, non‐financial support from Janssen Research and Development LLC for an MRC funded study led by PRAS, editorial honoraria and non‐financial support from Frontiers in Psychiatry outside the submitted work. He is a member of the UK Advisory Council on the Misuse of Drugs (ACMD), and Speciality Chief Editor, Mood Disorders section, Frontiers in Psychiatry. Eduard Vieta has received grants and served as consultant, advisor or CME speaker for the following entities (unrelated to the present work): AB‐Biotics, Abbott, Abbvie, Aimentia, Angelini, Biogen, Biohaven, Boehringer Ingelheim, Casen‐Recordati, Celon, Compass, Dainippon Sumitomo Pharma, Ethypharm, Ferrer, Gedeon Richter, GH Research, Glaxo Smith‐Kline, Idorsia, Janssen, Lundbeck, Novartis, Organon, Otsuka, Rovi, Sage, Sanofi‐Aventis, Sunovion, Takeda, and Viatris. Lakshmi N. Yatham reports speaker/consultant fees from Abbvie, Alkermes, DSP, Gedeon Richter, Intracellular Therapies, Merck, Otsuka, Sanofi, Sunovion, and grant funding from Allergan (now AbbVie), CIHR, and Dainippon Sumitomo outside the submitted work. Allan H. Young receives funding from the National Institute for Health and Care Research (NIHR) Maudsley Biomedical Research Centre at South London and Maudsley NHS Foundation Trust and King's College London. Lars Vedel Kessing reports speaker fees from Lundbeck and Teva. Ayal Schaffer reports speaker/consultant fees from Abbvie, Lundback and Otsuka. Jeff Zarp has received honoraria from Lundbeck. The remaining authors report no conflicts of interest. The views expressed are those of the author(s) and not necessarily those of the NIHR or the Department of Health and Social Care.

## Supporting information


**Table S1:** Additional methodological details of studies assessing baseline cognitive functioning as predictor.

## Data Availability

The data that support the findings of this study are available from the corresponding author upon reasonable request.

## References

[1] E. Bora , M. Yücel , and C. Pantelis , “Cognitive Impairment in Schizophrenia and Affective Psychoses: Implications for DSM‐V Criteria and Beyond,” Schizophrenia Bulletin 36, no. 1 (2010): 36–42. 19776206 10.1093/schbul/sbp094 PMC2800141

[2] S. K. Hill , J. L. Reilly , M. S. Harris , et al., “A Comparison of Neuropsychological Dysfunction in First‐Episode Psychosis Patients With Unipolar Depression, Bipolar Disorder, and Schizophrenia,” Schizophrenia Research 113, no. 2–3 (2009): 167–175. 19450952 10.1016/j.schres.2009.04.020 PMC2773205

[3] P. L. Rock , J. P. Roiser , W. J. Riedel , and A. D. Blackwell , “Cognitive Impairment in Depression: A Systematic Review and Meta‐Analysis,” Psychological Medicine 44, no. 10 (2014): 2029–2040. 24168753 10.1017/S0033291713002535

[4] G. Xu , K. Lin , D. Rao , et al., “Neuropsychological Performance in Bipolar I, Bipolar II and Unipolar Depression Patients: A Longitudinal, Naturalistic Study,” Journal of Affective Disorders 136, no. 3 (2012): 328–339. 22169253 10.1016/j.jad.2011.11.029

[5] C. Cotrena , L. D. Branco , F. M. Shansis , and R. P. Fonseca , “Executive Function Impairments in Depression and Bipolar Disorder: Association With Functional Impairment and Quality of Life,” Journal of Affective Disorders 190 (2016): 744–753. 26606718 10.1016/j.jad.2015.11.007

[6] Y. R. Smith , T. Love , C. C. Persad , A. Tkaczyk , T. E. Nichols , and J. K. Zubieta , “Impact of Combined Estradiol and Norethindrone Therapy on Visuospatial Working Memory Assessed by Functional Magnetic Resonance Imaging,” Journal of Clinical Endocrinology and Metabolism 91, no. 11 (2006): 4476–4481. 16912129 10.1210/jc.2006-0907 PMC1861832

[7] S. D. Stoddart , N. J. Craddock , and L. A. Jones , “Differentiation of Executive and Attention Impairments in Affective Illness,” Psychological Medicine 37, no. 11 (2007): 1613–1623. 17472760 10.1017/S0033291707000712

[8] M. J. Green , L. Girshkin , K. Kremerskothen , O. Watkeys , and Y. Quidé , “A Systematic Review of Studies Reporting Data‐Driven Cognitive Subtypes Across the Psychosis Spectrum,” Neuropsychology Review 30 (2020): 446–460. 31853717 10.1007/s11065-019-09422-7

[9] T. E. Van Rheenen , K. E. Lewandowski , E. J. Tan , et al., “Characterizing Cognitive Heterogeneity on the Schizophrenia‐Bipolar Disorder Spectrum,” Psychological Medicine 47, no. 10 (2017): 1848–1864. 28241891 10.1017/S0033291717000307

[10] C. Bourne , Ö. Aydemir , V. Balanzá‐Martínez , et al., “Neuropsychological Testing of Cognitive Impairment in Euthymic Bipolar Disorder: An Individual Patient Data Meta‐Analysis,” Acta Psychiatrica Scandinavica 128, no. 3 (2013): 149–162. 23617548 10.1111/acps.12133

[11] M. Semkovska , L. Quinlivan , T. O'Grady , et al., “Cognitive Function Following a Major Depressive Episode: A Systematic Review and Meta‐Analysis,” Lancet Psychiatry 6, no. 10 (2019): 851–861. 31422920 10.1016/S2215-0366(19)30291-3

[12] C. A. Depp , B. T. Mausbach , A. L. Harmell , et al., “Meta‐Analysis of the Association Between Cognitive Abilities and Everyday Functioning in Bipolar Disorder,” Bipolar Disorders 14, no. 3 (2012): 217–226. 22548895 10.1111/j.1399-5618.2012.01011.x PMC3396289

[13] R. S. McIntyre , D. S. Cha , J. K. Soczynska , et al., “Cognitive Deficits and Functional Outcomes in Major Depressive Disorder: Determinants, Substrates, and Treatment Interventions,” Depression and Anxiety 30, no. 6 (2013): 515–527. 23468126 10.1002/da.22063

[14] L. A. O'Donnell , P. J. Deldin , A. Grogan‐Kaylor , et al., “Depression and Executive Functioning Deficits Predict Poor Occupational Functioning in a Large Longitudinal Sample With Bipolar Disorder,” Journal of Affective Disorders 215 (2017): 135–142. 28319690 10.1016/j.jad.2017.03.015

[15] J. K. Tamura , D. Harangi , N. B. Rodrigues , et al., “The Mediational Role of Cognitive Function on Occupational Outcomes in Persons With Major Depressive and Bipolar Disorder,” CNS Spectrums 29, no. 6 (2024): 697–704. 39789718 10.1017/S1092852924002293

[16] S. Tse , S. Chan , K. L. Ng , and L. N. Yatham , “Meta‐Analysis of Predictors of Favorable Employment Outcomes Among Individuals With Bipolar Disorder,” Bipolar Disorders 16, no. 3 (2014): 217–229. 24219657 10.1111/bdi.12148

[17] C. Ribera , S. L. Vidal‐Rubio , J. E. Romeu‐Climent , J. Vila‐Francés , T. E. Van Rheenen , and V. Balanzá‐Martínez , “Cognitive Impairment and Consumption of Mental Healthcare Resources in Outpatients With Bipolar Disorder,” Journal of Psychiatric Research 138 (2021): 535–540. 33990024 10.1016/j.jpsychires.2021.05.003

[18] K. W. Miskowiak , J. Mariegaard , F. S. Jahn , and H. L. Kjærstad . “Associations Between Cognition and Subsequent Mood Episodes in Patients with Bipolar Disorder and Their Unaffected Relatives: A Systematic Review,” Journal of Affective Disorders 297 (2022): 176–188. 34699850 10.1016/j.jad.2021.10.044

[19] G. Perini , M. Cotta Ramusino , E. Sinforiani , S. Bernini , R. Petrachi , and A. Costa , “Cognitive Impairment in Depression: Recent Advances and Novel Treatments,” Neuropsychiatric Disease and Treatment 15 (2019): 1249–1258. 31190831 10.2147/NDT.S199746 PMC6520478

[20] A. Sankar , S. C. Ziersen , B. Ozenne , et al., “Association of Neurocognitive Function With Psychiatric Hospitalization and Socio‐Demographic Conditions in Individuals With Bipolar and Major Depressive Disorders,” EClinicalMedicine 58 (2023): 101927. 37007740 10.1016/j.eclinm.2023.101927 PMC10050788

[21] S. J. Groves , K. M. Douglas , W. Moot , et al., “Relationship Between Baseline Cognition and 18‐Month Treatment Response in Bipolar Disorder,” Journal of Affective Disorders 318 (2022): 224–230. 36055530 10.1016/j.jad.2022.08.112

[22] I. Seeberg , H. L. Kjaerstad , and K. W. Miskowiak , “Neural and Behavioral Predictors of Treatment Efficacy on Mood Symptoms and Cognition in Mood Disorders: A Systematic Review,” Frontiers in Psychiatry 9 (2018): 337. 30093870 10.3389/fpsyt.2018.00337 PMC6071514

[23] S. Brissos , V. V. Dias , A. I. Carita , and A. Martinez‐Arán , “Quality of Life in Bipolar Type I Disorder and Schizophrenia in Remission: Clinical and Neurocognitive Correlates,” Psychiatry Research 160, no. 1 (2008): 55–62. 18485488 10.1016/j.psychres.2007.04.010

[24] M. J. Knight , E. Lyrtzis , and B. T. Baune , “The Association of Cognitive Deficits With Mental and Physical Quality of Life in Major Depressive Disorder,” Comprehensive Psychiatry 97 (2020): 152147. 31838296 10.1016/j.comppsych.2019.152147

[25] K. W. Miskowiak , A. F. Carvalho , E. Vieta , and L. V. Kessing , “Cognitive Enhancement Treatments for Bipolar Disorder: A Systematic Review and Methodological Recommendations,” European Neuropsychopharmacology: The Journal of the European College of Neuropsychopharmacology 26, no. 10 (2016): 1541–1561. 27593623 10.1016/j.euroneuro.2016.08.011

[26] K. W. Miskowiak , C. V. Ott , J. Z. Petersen , and L. V. Kessing , “Systematic Review of Randomized Controlled Trials of Candidate Treatments for Cognitive Impairment in Depression and Methodological Challenges in the Field,” European Neuropsychopharmacology: The Journal of the European College of Neuropsychopharmacology 26, no. 12 (2016): 1845–1867. 27745932 10.1016/j.euroneuro.2016.09.641

[27] K. W. Miskowiak , I. Seeberg , M. B. Jensen , et al., “Randomised Controlled Cognition Trials in Remitted Patients With Mood Disorders Published Between 2015 and 2021: A Systematic Review by the International Society for Bipolar Disorders Targeting Cognition Task Force,” Bipolar Disorders 24, no. 4 (2022): 354–374. 35174594 10.1111/bdi.13193 PMC9541874

[28] K. M. Douglas and T. E. Van Rheenen , “Current Treatment Options for Cognitive Impairment in Bipolar Disorder: A Review,” Current Treatment Options in Psychiatry 3 (2016): 330–355.

[29] J. K. Tamura , I. P. Carvalho , L. M. W. Leanna , et al., “Management of Cognitive Impairment in Bipolar Disorder: A Systematic Review of Randomized Controlled Trials,” CNS Spectrums 27, no. 4 (2022): 399–420. 10.1017/S1092852921000092 33706820

[30] K. W. Miskowiak , K. E. Burdick , A. Martinez‐Aran , et al., “Methodological Recommendations for Cognition Trials in Bipolar Disorder by the International Society for Bipolar Disorders Targeting Cognition Task Force,” Bipolar Disorders 19, no. 8 (2017): 614–626. 28895274 10.1111/bdi.12534 PMC6282834

[31] M. J. Page , J. E. McKenzie , P. M. Bossuyt , et al., “The PRISMA 2020 Statement: An Updated Guideline for Reporting Systematic Reviews,” BMJ 372 (2021): n71. 33782057 10.1136/bmj.n71 PMC8005924

[32] American Psychiatric Association , Diagnostic and Statistical Manual of Mental Disorders: DSM‐5, 5th ed. (American Psychiatric Publishing, 2013).

[33] World Health Organization , “ICD‐11: International Classification of Diseases (11th Revision),” (2022), https://icd.who.int/.

[34] M. Ouzzani , H. Hammady , Z. Fedorowicz , and A. Elmagarmid , “Rayyan—A Web and Mobile App for Systematic Reviews,” Systematic Reviews 5 (2016): 1–10. 27919275 10.1186/s13643-016-0384-4 PMC5139140

[35] J. A. C. Sterne , J. Savović , M. J. Page , et al., “RoB 2: A Revised Tool for Assessing Risk of Bias in Randomised Trials,” BMJ (Clinical Research Ed.) 366 (2019): l4898. 10.1136/bmj.l4898 31462531

[36] B. T. Baune , E. Sampson , J. Louise , et al., “No Evidence for Clinical Efficacy of Adjunctive Celecoxib With Vortioxetine in the Treatment of Depression: A 6‐Week Double‐Blind Placebo Controlled Randomized Trial,” European Neuropsychopharmacology 53 (2021): 34–46. 34375789 10.1016/j.euroneuro.2021.07.092

[37] K. E. Burdick , R. J. Braga , C. U. Nnadi , Y. Shaya , W. H. Stearns , and A. K. Malhotra , “Placebo‐Controlled Adjunctive Trial of Pramipexole in Patients With Bipolar Disorder: Targeting Cognitive Dysfunction,” Journal of Clinical Psychiatry 73, no. 1 (2012): 103–112. 22152405 10.4088/JCP.11m07299 PMC4457389

[38] O. M. Dean , A. I. Bush , D. L. Copolov , et al., “Effects of N‐Acetyl Cysteine on Cognitive Function in Bipolar Disorder,” Psychiatry and Clinical Neurosciences 66, no. 6 (2012): 514–517. 23066769 10.1111/j.1440-1819.2012.02392.x

[39] D. P. Devanand , G. H. Pelton , K. D'Antonio , et al., “Donepezil Treatment in Patients With Depression and Cognitive Impairment on Stable Antidepressant Treatment: A Randomized Controlled Trial,” American Journal of Geriatric Psychiatry 26, no. 10 (2018): 1050–1060. 10.1016/j.jagp.2018.05.008 PMC6396676 30037778

[40] M. Kaser , J. B. Deakin , A. Michael , et al., “Modafinil Improves Episodic Memory and Working Memory Cognition in Patients With Remitted Depression: A Double‐Blind, Randomized, Placebo‐Controlled Study,” Biological Psychiatry: Cognitive Neuroscience and Neuroimaging 2, no. 2 (2017): 115–122. 28299368 10.1016/j.bpsc.2016.11.009 PMC5339412

[41] B. Krause‐Sorio , P. Siddarth , K. T. Laird , et al., “[18F] FDDNP PET Binding Predicts Change in Executive Function in a Pilot Clinical Trial of Geriatric Depression,” International Psychogeriatrics 33, no. 2 (2021): 149–156. 31969201 10.1017/S1041610219002047 PMC7375908

[42] J. Macoveanu , J. Z. Petersen , J. Mariegaard , et al., “Effects of Erythropoietin on Cognitive Impairment and Prefrontal Cortex Activity Across Affective Disorders: A Randomized, Double‐Blinded, Placebo‐Controlled Trial,” Journal of Psychopharmacology 38, no. 4 (2024): 362–374. 38519416 10.1177/02698811241237869

[43] R. S. McIntyre , I. Florea , B. Tonnoir , H. Loft , R. W. Lam , and M. C. Christensen , “Efficacy of Vortioxetine on Cognitive Functioning in Working Patients With Major Depressive Disorder,” Journal of Clinical Psychiatry 78, no. 1 (2017): 22276. 10.4088/JCP.16m10744 27780334

[44] K. W. Miskowiak , J. L. Forman , M. Vinberg , H. R. Siebner , L. V. Kessing , and J. Macoveanu , “Impact of Pretreatment Interhemispheric Hippocampal Asymmetry on Improvement in Verbal Learning Following Erythropoietin Treatment in Mood Disorders: A Randomized Controlled Trial,” Journal of Psychiatry and Neuroscience 45, no. 3 (2020): 198–205. 31804779 10.1503/jpn.180205 PMC7828975

[45] K. W. Miskowiak , A. J. Rush , T. A. Gerds , M. Vinberg , and L. V. Kessing , “Targeting Treatments to Improve Cognitive Function in Mood Disorder: Suggestions From Trials Using Erythropoietin,” Journal of Clinical Psychiatry 77, no. 12 (2016): 6577. 10.4088/JCP.15m10480 27835716

[46] G. M. Murphy, Jr. , J. E. Sarginson , H. S. Ryan , R. O'Hara , A. F. Schatzberg , and L. C. Lazzeroni , “BDNF and CREB1 Genetic Variants Interact to Affect Antidepressant Treatment Outcomes in Geriatric Depression,” Pharmacogenetics and Genomics 23, no. 6 (2013): 301–313. 23619509 10.1097/FPC.0b013e328360b175

[47] I. J. Osuji , E. Vera‐Bolaños , T. J. Carmody , and E. S. Brown , “Pregnenolone for Cognition and Mood in Dual Diagnosis Patients,” Psychiatry Research 178, no. 2 (2010): 309–312. 20493557 10.1016/j.psychres.2009.09.006

[48] C. V. Ott , M. Vinberg , L. V. Kessing , and K. W. Miskowiak , “The Effect of Erythropoietin on Cognition in Affective Disorders–Associations With Baseline Deficits and Change in Subjective Cognitive Complaints,” European Neuropsychopharmacology 26, no. 8 (2016): 1264–1273. 27349944 10.1016/j.euroneuro.2016.05.009

[49] G. H. Pelton , L. Soleimani , S. P. Roose , M. H. Tabert , and D. P. Devanand , “Olfactory Deficits Predict Cognitive Improvement on Donepezil in Patients With Depression and Cognitive Impairment: A Randomized Controlled Pilot Study,” Alzheimer Disease and Associated Disorders 30, no. 1 (2016): 67–69. 26398910 10.1097/WAD.0000000000000107 PMC4764438

[50] J. K. Soczynska , L. N. Ravindran , R. Styra , et al., “The Effect of Bupropion XL and Escitalopram on Memory and Functional Outcomes in Adults With Major Depressive Disorder: Results From a Randomized Controlled Trial,” Psychiatry Research 220, no. 1–2 (2014): 245–250. 25124683 10.1016/j.psychres.2014.06.053

[51] K. Van Dyk , P. Siddarth , M. Rossetti , L. M. Ercoli , M. M. Milillo , and H. Lavretsky , “Memantine Can Protect Against Inflammation‐Based Cognitive Decline in Geriatric Depression,” Brain, Behavior, and Immunity‐Health 9 (2020): 100167. 10.1016/j.bbih.2020.100167 PMC8474499 34589902

[52] A. R. Van Meter , M. M. Perez‐Rodriguez , R. J. Braga , et al., “Pramipexole to Improve Cognition in Bipolar Disorder: A Randomized Controlled Trial,” Journal of Clinical Psychopharmacology 41, no. 4 (2021): 421–427. 33956703 10.1097/JCP.0000000000001407 PMC8238822

[53] S. Watson , P. Gallagher , R. J. Porter , et al., “A Randomized Trial to Examine the Effect of Mifepristone on Neuropsychological Performance and Mood in Patients With Bipolar Depression,” Biological Psychiatry 72, no. 11 (2012): 943–949. 22770649 10.1016/j.biopsych.2012.05.029

[54] L. Xue , M. Bocharova , A. H. Young , and D. Aarsland , “Cognitive Improvement in Late‐Life Depression Treated With Vortioxetine and Duloxetine in an Eight‐Week Randomized Controlled Trial: The Role of Age at First Onset and Change in Depressive Symptoms,” Journal of Affective Disorders 361 (2024): 74–81. 38838790 10.1016/j.jad.2024.06.003

[55] A. H. Young , P. Gallagher , S. Watson , D. Del‐Estal , B. M. Owen , and I. Nicol Ferrier , “Improvements in Neurocognitive Function and Mood Following Adjunctive Treatment With Mifepristone (RU‐486) in Bipolar Disorder,” Neuropsychopharmacology 29, no. 8 (2004): 1538–1545. 15127079 10.1038/sj.npp.1300471

[56] C. D. M. Bonnin , M. Reinares , A. Martínez‐Arán , et al., “Effects of Functional Remediation on Neurocognitively Impaired Bipolar Patients: Enhancement of Verbal Memory,” Psychological Medicine 46, no. 2 (2016): 291–301. 26387583 10.1017/S0033291715001713

[57] K. M. Demant , M. Vinberg , L. V. Kessing , and K. W. Miskowiak , “Effects of Short‐Term Cognitive Remediation on Cognitive Dysfunction in Partially or Fully Remitted Individuals With Bipolar Disorder: Results of a Randomised Controlled Trial,” PLoS One 10, no. 6 (2015): e0127955. 26070195 10.1371/journal.pone.0127955 PMC4467086

[58] K. M. Douglas , S. Groves , M. T. Crowe , et al., “A Randomised Controlled Trial of Psychotherapy and Cognitive Remediation to Target Cognition in Mood Disorders,” Acta Psychiatrica Scandinavica 145, no. 3 (2022): 278–292. 34800298 10.1111/acps.13387

[59] A. E. Jespersen , I. S. Røen , A. Lumbye , M. Nordentoft , L. B. Glenthøj , and K. W. Miskowiak , “Feasibility and Effect of an Immersive Virtual Reality‐Based Platform for Cognitive Training in Real‐Life Scenarios in Patients With Mood‐Or Psychotic Disorders: A Randomized, Controlled Proof‐Of‐Concept Study,” Neuroscience Applied 2 (2023): 101120. 40655976 10.1016/j.nsa.2023.101120 PMC12244015

[60] L. Listunova , M. Bartolovic , J. Kienzle , et al., “Predictors of Cognitive Remediation Therapy Improvement in (Partially) Remitted Unipolar Depression,” Journal of Affective Disorders 264 (2020): 40–49. 31846901 10.1016/j.jad.2019.12.006

[61] K. W. Miskowiak , A. B. Møller , and C. V. Ott , “Neuronal and Cognitive Predictors of Improved Executive Function Following Action‐Based Cognitive Remediation in Patients With Bipolar Disorder,” European Neuropsychopharmacology 47 (2021): 1–10. 33725651 10.1016/j.euroneuro.2021.02.013

[62] M. B. Mogensen , J. Macoveanu , G. M. Knudsen , C. V. Ott , and K. W. Miskowiak , “Influence of Pre‐Treatment Structural Brain Measures on Effects of Action‐Based Cognitive Remediation on Executive Function in Partially or Fully Remitted Patients With Bipolar Disorder,” European Neuropsychopharmacology 56 (2022): 50–59. 34933219 10.1016/j.euroneuro.2021.11.010

[63] D. Primavera , C. Aviles Gonzalez , A. Perra , et al., “Virtual Reality Cognitive Remediation in Older Adults With Bipolar Disorder: The Effects on Cognitive Performance and Depression in a Feasibility Randomized Controlled Trial,” Health 12, no. 17 (2024): 1753. 10.3390/healthcare12171753 PMC11394966 39273777

[64] J. Sanchez‐Moreno , C. Bonnín , A. González‐Pinto , et al., “Do Patients With Bipolar Disorder and Subsyndromal Symptoms Benefit From Functional Remediation? A 12‐Month Follow‐Up Study,” European Neuropsychopharmacology 27, no. 4 (2017): 350–359. 28126401 10.1016/j.euroneuro.2017.01.010

[65] M. Semkovska and E. Ahern , “Online Neurocognitive Remediation Therapy to Improve Cognition in Community‐Living Individuals With a History of Depression: A Pilot Study,” Internet Interventions 9 (2017): 7–14. 30135832 10.1016/j.invent.2017.04.003 PMC6096308

[66] B. Solé , C. M. Bonnin , M. Mayoral , et al., “Functional Remediation for Patients With Bipolar II Disorder: Improvement of Functioning and Subsyndromal Symptoms,” European Neuropsychopharmacology 25, no. 2 (2015): 257–264. 24906790 10.1016/j.euroneuro.2014.05.010

[67] R. Strawbridge , D. Tsapekos , and A. H. Young , “Circulating Inflammatory and Neurotrophic Markers as Moderators and/or Mediators of Cognitive Remediation Outcome in People With Bipolar Disorders,” BJPsych Open 10, no. 6 (2024): e225. 39635758 10.1192/bjo.2024.818 PMC11698213

[68] K. R. Thomas , O. Puig , and E. W. Twamley , “Age as a Moderator of Change Following Compensatory Cognitive Training in Individuals With Severe Mental Illnesses,” Psychiatric Rehabilitation Journal 40, no. 1 (2017): 70–78. 27547856 10.1037/prj0000206 PMC5322254

[69] D. Tsapekos , R. Strawbridge , M. Cella , T. Wykes , and A. H. Young , “Towards Personalizing Cognitive Remediation Therapy: Examining Moderators of Response for Euthymic People With Bipolar Disorder,” Behaviour Research and Therapy 151 (2022): 104054. 35168010 10.1016/j.brat.2022.104054

[70] E. W. Twamley , K. R. Thomas , C. Z. Burton , et al., “Compensatory Cognitive Training for People With Severe Mental Illnesses in Supported Employment: A Randomized Controlled Trial,” Schizophrenia Research 203 (2019): 41–48. 28823720 10.1016/j.schres.2017.08.005 PMC5816728

[71] S. Wanmaker , E. Geraerts , and I. H. Franken , “A Working Memory Training to Decrease Rumination in Depressed and Anxious Individuals: A Double‐Blind Randomized Controlled Trial,” Journal of Affective Disorders 175 (2015): 310–319. 25661397 10.1016/j.jad.2014.12.027

[72] S. M. McClintock , D. M. Martin , S. H. Lisanby , et al., “Neurocognitive Effects of Transcranial Direct Current Stimulation (tDCS) in Unipolar and Bipolar Depression: Findings From an International Randomized Controlled Trial,” Depression and Anxiety 37, no. 3 (2020): 261–272. 31944487 10.1002/da.22988

[73] S. E. Nadeau , D. Bowers , T. L. Jones , S. S. Wu , W. J. Triggs , and K. M. Heilman , “Cognitive Effects of Treatment of Depression With Repetitive Transcranial Magnetic Stimulation,” Cognitive and Behavioral Neurology 27, no. 2 (2014): 77–87. 24968008 10.1097/WNN.0000000000000031

[74] V. Buschert , D. Prochazka , H. Bartl , et al., “Effects of Physical Activity on Cognitive Performance: A Controlled Clinical Study in Depressive Patients,” European Archives of Psychiatry and Clinical Neuroscience 269 (2019): 555–563. 29951850 10.1007/s00406-018-0916-0

[75] B. M. Hoffman , J. A. Blumenthal , M. A. Babyak , et al., “Exercise Fails to Improve Neurocognition in Depressed Middle‐Aged and Older Adults,” Medicine and Science in Sports and Exercise 40, no. 7 (2008): 1344–1352. 18580416 10.1249/MSS.0b013e31816b877c PMC2745928

[76] B. Seccomandi , D. Tsapekos , K. Newbery , T. Wykes , and M. Cella , “A Systematic Review of Moderators of Cognitive Remediation Response for People With Schizophrenia,” Schizophrenia Research: Cognition 19 (2020): 100160. 31828023 10.1016/j.scog.2019.100160 PMC6889639

[77] R. J. Jutten , E. Grandoit , N. S. Foldi , et al., “Lower Practice Effects as a Marker of Cognitive Performance and Dementia Risk: A Literature Review,” Alzheimer's & Dementia 12, no. 1 (2020): e12055. 10.1002/dad2.12055 PMC7346865 32671181

[78] L. N. Yatham , I. J. Torres , G. S. Malhi , et al., “The International Society for Bipolar Disorders–Battery for Assessment of Neurocognition (ISBD‐BANC),” Bipolar Disorders 12, no. 4 (2010): 351–363. 20636632 10.1111/j.1399-5618.2010.00830.x

[79] L. N. Yatham , S. Mackala , J. Basivireddy , et al., “Lurasidone Versus Treatment as Usual for Cognitive Impairment in Euthymic Patients With Bipolar I Disorder: A Randomised, Open‐Label, Pilot Study,” Lancet Psychiatry 4, no. 3 (2017): 208–217. 28185899 10.1016/S2215-0366(17)30046-9

[80] S. E. Purdon and R. Psych , The Screen for Cognitive Impairment in Psychiatry. Administration and Psychometric Properties (PNL, 2005).

[81] E. Bora , “A Meta‐Analysis of Data‐Driven Cognitive Subgroups in Bipolar Disorder,” European Neuropsychopharmacology 90 (2025): 48–57. 39509830 10.1016/j.euroneuro.2024.10.008

[82] B. Solé and D. Clougher , “Cognitive Screening in Bipolar Disorder,” European Neuropsychopharmacology: The Journal of the European College of Neuropsychopharmacology 85 (2024): 1. 38643626 10.1016/j.euroneuro.2024.04.008

[83] D. Tsapekos , R. Strawbridge , M. Cella , T. Wykes , and A. H. Young , “Cognitive Impairment in Euthymic Patients With Bipolar Disorder: Prevalence Estimation and Model Selection for Predictors of Cognitive Performance,” Journal of Affective Disorders 294 (2021): 497–504. 34330045 10.1016/j.jad.2021.07.036

[84] J. R. Blair and O. Spreen , “Predicting Premorbid IQ: A Revision of the National Adult Reading Test,” Clinical Neuropsychologist 3, no. 2 (1989): 129–136.

[85] D. Wechsler , Test of Premorbid Functioning. UK Version (TOPF UK) (Pearson. Inc., 2011).

[86] D. Tsapekos , M. Kalfas , R. Strawbridge , S. Swidzinski , K. E. Burdick , and A. H. Young , “Estimating Cognitive Impairment in Bipolar Disorder: Should We Account for Premorbid IQ?,” Acta Psychiatrica Scandinavica 153, no. 5 (2026): 468–476.

[87] J. Z. Petersen , R. J. Porter , and K. W. Miskowiak , “Clinical Characteristics Associated With the Discrepancy Between Subjective and Objective Cognitive Impairment in Depression,” Journal of Affective Disorders 246 (2019): 763–774. 30623822 10.1016/j.jad.2018.12.105

[88] I. J. Torres , S. Mackala , S. Ahn , et al., “Relationship Between Subjective Cognitive Functioning and Fluid and Crystallized Cognitive Abilities in Bipolar Disorder,” Journal of the International Neuropsychological Society 30, no. 8 (2024): 719–727. 39410816 10.1017/S1355617724000559

[89] M. De Prisco and E. Vieta , “The Never‐Ending Problem: Sample Size Matters,” European Neuropsychopharmacology: The Journal of the European College of Neuropsychopharmacology 79 (2024): 17–18. 38056029 10.1016/j.euroneuro.2023.10.002

[90] A. G. Barnett , J. C. van der Pols , and A. J. Dobson , “Regression to the Mean: What It Is and How to Deal With It,” International Journal of Epidemiology 34, no. 1 (2005): 215–220. 15333621 10.1093/ije/dyh299

[91] R. M. Baron and D. A. Kenny , “The Moderator–Mediator Variable Distinction in Social Psychological Research: Conceptual, Strategic, and Statistical Considerations,” Journal of Personality and Social Psychology 51, no. 6 (1986): 1173–1182. 3806354 10.1037//0022-3514.51.6.1173

[92] H. C. Kraemer , “Messages for Clinicians: Moderators and Mediators of Treatment Outcome in Randomized Clinical Trials,” American Journal of Psychiatry 173, no. 7 (2016): 672–679. 26988629 10.1176/appi.ajp.2016.15101333

[93] L. Ilzarbe and E. Vieta , “The Elephant in the Room: Medication as Confounder,” European Neuropsychopharmacology: The Journal of the European College of Neuropsychopharmacology 71 (2023): 6–8. 36931073 10.1016/j.euroneuro.2023.03.001

[94] A. Mlinarić , M. Horvat , and V. Šupak Smolčić , “Dealing With the Positive Publication Bias: Why You Should Really Publish Your Negative Results,” Biochemia Medica 27, no. 3 (2017): 030201. 29180912 10.11613/BM.2017.030201 PMC5696751

[95] K. W. Miskowiak , T. K. Roikjer , J. Mariegaard , et al., “Implementing Cognitive Screenings for Outpatients With Bipolar Disorder Following Optimised Treatment in a Specialised Mood Disorder Clinic,” European Neuropsychopharmacology: The Journal of the European College of Neuropsychopharmacology 84 (2024): 27–34. 38643698 10.1016/j.euroneuro.2024.04.013

