# Supplementary material for: Predicting Response to Pro‐Cognitive Interventions in Mood Disorders: A Systematic Review by the International Society for Bipolar Disorders Targeting Cognition Task Force
Source: Acta Psychiatr Scand. 2025 Oct 13;153(5):374–401. doi: 10.1111/acps.70038 (PMC13050603; doi:10.1111/acps.70038)
Supplement: Supplementary file 1 — Table S1: Additional methodological details of studies assessing baseline cognitive functioning as predictor. [file ACPS-153-374-s001.docx]

| **Table S1**. Additional methodological details of studies assessing baseline cognitive functioning as predictor | | | | |
| --- | --- | --- | --- | --- |
| **Study** | **Baseline Objective Cognitive Impairment** | | | **Analysis** |
|  | ***Cognitively impaired sample?*** | ***Definition of cognitive impairment*** | ***Impairment measure used as outcome?*** |  |
| Bonnin 2016 | Yes | ≥2 SDs below norms on ≥1 test | Yes, CVLT among multiple tests used for impairment classification | ANOVA  (verbal episodic memory scores at baseline vs follow-up in the cognitively impaired subsample) |
| Devanand 2018 | Yes | EMCI: MMSE≥21 and ≤11 for delayed recall on WMS-RLM II or ≥1.5 SDs below norms for delayed recall on SRT  LMCI: ≥1.5 SDs below norms on WMS Revised Logical Memory or SRT | SRT (yes) / ADAS-Cog (no) | Linear mixed models  (baseline cognitive impairment as predictor of change in ADAS-Cog or SRT) |
| Jespersen 2023 | No | N/A | N/A | Correlation  (baseline CAVIR and CAVIR change score in the treatment group) |
| Kaser 2017 | No | N/A | N/A | Non-parametric correlations  (between baseline and post-treatment scores (i.e., not cognitive change scores) |
| Listunova 2020 | Yes | Scores below PR of 16 in at least two cognitive tests (VTS, SCT, CVLT) from a selection of six cognitive subdomains | Yes | T test  (differences in multiple cognitive domains at baseline between improvers vs non-improvers) |
| Miskowiak 2016c | No | RAVLT ≤1SD below norms | Yes, RAVLT but alternative versions | Logistic regression (memory dysfunction at baseline as predictor of clinically relevant EPO-associated memory improvement) |
| Miskowiak 2021 | Yes | SCIP ≤74, or below defined cut-off on ≥2 subtests | No | Linear regression (baseline executive dysfunction as predictor of post-treatment improvement in executive functioning) |
| Nadeau 2014 | No | N/A | N/A | Linear regression (baseline executive functioning as predictor of post-treatment improvement in executive functioning) |
| Ott 2016 | No | ≥1 SD below norms on ≥2 tests (RAVLT, RBANS, verbal fluency, WAIS-III letter-number sequencing, TMT-B, RVP from CANTAB) | Yes, improvement of ≥1 SD on  ≥2 tests | Logistic regression (baseline cognitive dysfunction as predictor of clinically relevant post-treatment cognitive improvement) |
| Tsapekos 2022 | No | N/A | N/A | Linear regression  (baseline cognitive performance as predictor of change in verbal memory) |
| *Notes:* ADAS-Cog: Alzheimer’s Disease Assessment Scale—Cognitive subscale; CANTAB: Cambridge Neuropsychological Test Automated Battery; CAVIR: Cognition Assessment in Virtual Reality; CR: Cognitive Remediation; CVLT: California Verbal Learning Test; EMCI: Early Mild Cognitive Impairment; LMCI: Late Mild Cognitive Impairment; MMSE: Folstein Mini-Mental State Exam; N/A: Not Applicable; PR: Percentile Rank; RAVLT: Rey Auditory Verbal Learning Test; RBANS: Repeatable Battery for the Assessment of Neuropsychological Status; RLM: Revised Logical Memory; RVP: Rapid Visual Information Processing; SCT: Symbol Coding Task; SCIP: Screen for Cognitive Impairment in Psychiatry; SD: Standard Deviation; SRT: Selective Reminding Test; TMT-B: Trail Making Test-B; VTS: Vienna Test System; WAIS: Wechsler Adult Intelligence Scale; WMS: Wechsler Memory Scale. | | | | |
